# CCR5 antagonist reduces HIV-induced amyloidogenesis, tau pathology, neurodegeneration, and blood-brain barrier alterations in HIV-infected hu-PBL-NSG mice

**DOI:** 10.1186/s13024-021-00500-0

**Published:** 2021-11-22

**Authors:** Biju Bhargavan, Shawna M. Woollard, Jo Ellyn McMillan, Georgette D. Kanmogne

**Affiliations:** 1 Department of Pharmacology and Experimental Neuroscience, College of Medicine, University of Nebraska Medical Center, 985800 Nebraska Medical Center, Omaha, NE 68198-5800 USA; 2 Huvepharma, 421 W Industrial Lake Drive, Lincoln, NE 68528 USA

**Keywords:** HIV-1, NSG mice, Amyloid-beta, Tau phosphorylation, Neuronal damage, Blood-brain barrier injury, CCR5, Maraviroc, Monocytes-derived macrophages, Human brain microvascular endothelial cells, RAGE, LRP1

## Abstract

**Background:**

Neurocognitive impairment is present in 50% of HIV-infected individuals and is often associated with Alzheimer’s Disease (AD)-like brain pathologies, including increased amyloid-beta (Aβ) and Tau hyperphosphorylation. Here, we aimed to determine whether HIV-1 infection causes AD-like pathologies in an HIV/AIDS humanized mouse model, and whether the CCR5 antagonist maraviroc alters HIV-induced pathologies.

**Methods:**

NOD/scid–IL-2Rγ_c_^null^ mice engrafted with human blood leukocytes were infected with HIV-1, left untreated or treated with maraviroc (120 mg/kg twice/day). Human cells in animal’s blood were quantified weekly by flow cytometry. Animals were sacrificed at week-3 post-infection; blood and tissues viral loads were quantified using p24 antigen ELISA, RNAscope, and qPCR. Human (HLA-DR+) cells, Aβ-42, phospho-Tau, neuronal markers (MAP 2, NeuN, neurofilament-L), gamma-secretase activating protein (GSAP), and blood-brain barrier (BBB) tight junction (TJ) proteins expression and transcription were quantified in brain tissues by immunohistochemistry, immunofluorescence, immunoblotting, and qPCR. Plasma Aβ-42, Aβ-42 cellular uptake, release and transendothelial transport were quantified by ELISA.

**Results:**

HIV-1 significantly decreased human (h)CD4+ T-cells and hCD4/hCD8 ratios; decreased the expression of BBB TJ proteins claudin-5, ZO-1, ZO-2; and increased HLA-DR+ cells in brain tissues. Significantly, HIV-infected animals showed increased plasma and brain Aβ-42 and phospho-Tau (threonine181, threonine231, serine396, serine199), associated with transcriptional upregulation of GSAP, an enzyme that catalyzes Aβ formation, and loss of MAP 2, NeuN, and neurofilament-L. Maraviroc treatment significantly reduced blood and brain viral loads, prevented HIV-induced loss of neuronal markers and TJ proteins; decreased HLA-DR+ cells infiltration in brain tissues, significantly reduced HIV-induced increase in Aβ-42, GSAP, and phospho-Tau. Maraviroc also reduced Aβ retention and increased Aβ release in human macrophages; decreased the receptor for advanced glycation end products (RAGE) and increased low-density lipoprotein receptor–related protein-1 (LRP1) expression in human brain endothelial cells. Maraviroc induced Aβ transendothelial transport, which was blocked by LRP1 antagonist but not RAGE antagonist.

**Conclusions:**

Maraviroc significantly reduced HIV-induced amyloidogenesis, GSAP, phospho-Tau, neurodegeneration, BBB alterations, and leukocytes infiltration into the CNS. Maraviroc increased cellular Aβ efflux and transendothelial Aβ transport via LRP1 pathways. Thus, therapeutically targeting CCR5 could reduce viremia, preserve the BBB and neurons, increased brain Aβ efflux, and reduce AD-like neuropathologies.

**Supplementary Information:**

The online version contains supplementary material available at 10.1186/s13024-021-00500-0.

## Background

The human immunodeficiency virus-1 (HIV-1) enters target cells by binding its envelope glycoprotein gp160 to the CD4 receptor and/or coreceptors such as the C-C chemokine receptor type-5 (CCR5) and C-X-C chemokine receptor type-4 (CXCR4) [1]. CCR5- and CXCR4-tropic viral strains use CCR5 and CXCR4, respectively, as their coreceptor to enter and infect target cells; whereas some HIV strains are dual-tropic and can use CCR5 and/or CXCR4 [1]. CCR5 is expressed on several cell types, including brain endothelial cells [2], T-cells, dendritic cells, and leukocytes [3, 4]. In HIV-infected humans, CCR5-tropic viruses predominate during the early stages of infection, whereas CXCR4-tropic viruses usually emerge during the later stages [3, 4]. The importance of CCR5 in HIV infection and acquired immunodeficiency syndrome (AIDS) pathology was demonstrated by studies showing that a 32-base-pair deletion in the CCR5 gene resulted in resistance to HIV-1 infection or slower progression to AIDS [5, 6]. Given the importance of CCR5 in HIV-1 transmission, infection, and disease progression, this chemokine receptor has been a major therapeutic target for HIV/AIDS prevention and treatment. Maraviroc (MVC, Selzentry, ViiV Healthcare) is a small-molecule CCR5 antagonist with favorable safety, pharmacokinetic, and pharmacodynamic profiles [7, 8] that is FDA-approved for the treatment of CCR5-tropic HIV infection in both antiretroviral therapy (ART)-naïve and treatment-experienced patients [9, 10].

Following infection, HIV induces blood-brain barrier (BBB) injury, enters the central nervous system (CNS), and productively infects brain macrophages and glial cells [11–13]. This infection of CNS cells, production and release of virions and viral proteins into the brain, as well as subsequent increased inflammation and oxidative stress, results in neuronal injury and death [13–15]. These brain pathologies frequently result in behavioral, motor, and cognitive abnormalities referred to as HIV-associated neurocognitive disorders (HAND) [13, 15, 16]. Although the prevalence of HIV-associated dementia, the most severe form of HAND, has declined in the current ART era, milder forms of HAND [asymptomatic neurocognitive impairment and mild neurocognitive disorders] are still highly prevalent and occur in up to 50% of HIV-infected persons [13, 15, 16].

The molecular mechanisms associated with the development of HAND have not been well elucidated. Autopsy studies of HIV-infected people, including those who had been on long-term ART, showed the presence of proteopathy and Alzheimer’s Disease (AD)-like CNS pathologies, including increased deposits of amyloid-beta (Aβ), formation of amyloid plaques in neuronal cells and perivascular areas, hyperphosphorylation of Tau proteins, and the presence of neurofibrillary tangles (NFTs)-like structures [17–20]. The presence of Aβ deposits and Tau hyperphosphorylation in brain tissues of HIV-infected persons is often associated with high viral loads [21] and neurocognitive impairments, including impairments in speed of information processing, attention, and working memory [21, 22]. For the current study, our objective was to determine whether AD-like pathologies occur in a humanized mouse model of HIV/AIDS [NOD/*scid*–IL-2Rγ_c_^*null*^ mice engrafted with human blood leukocytes (hu-PBL-NSG)] and to assess the effects of the CCR5 antagonist MVC on HIV-induced brain pathologies in vivo. We have reproduced AD-like pathologies in this animal model. We demonstrate increased phosphorylation of Tau [at threonine (Thr)181, Thr231, serine (Ser)396, and Ser199)], and increased production and accumulation of Aβ in brain tissues and plasma of HIV-infected animals associated with transcriptional upregulation of gamma-secretase activating protein (GSAP), an endoprotease that catalyzes γ-secretase cleavage of amyloid precursor proteins (APP) and Aβ formation [23–26]. Most significantly, we have demonstrated that in addition to preserving the immune system and decreasing systemic and brain viral loads, the CCR5 antagonist MVC reduced HIV-induced BBB alterations and infiltration of leukocytes into the brain of infected animals, and significantly reduced HIV-induced neuronal injury, CNS Aβ formation, and Tau phosphorylation. Additional studies showed that MVC increased plasma Aβ levels, reduced Aβ retention and increased Aβ release in primary human macrophages; decreased brain endothelial expression of the receptor for advanced glycation end products (RAGE), an influx receptor that binds and transports circulating plasma Aβ into the CNS [27–30]; increased brain endothelial expression of the low-density lipoprotein receptor–related protein-1 (LRP1), an efflux-clearance receptor that binds and transports brain-derived Aβ into the blood [31–33]; and increased transendothelial Aβ transport via LRP1. These data suggest that therapeutically targeting CCR5 can reduce or abrogate HIV-induced AD-like neuropathologies.

## Methods

### Hu-PBL-NSG mice model

**Fig. 1:**
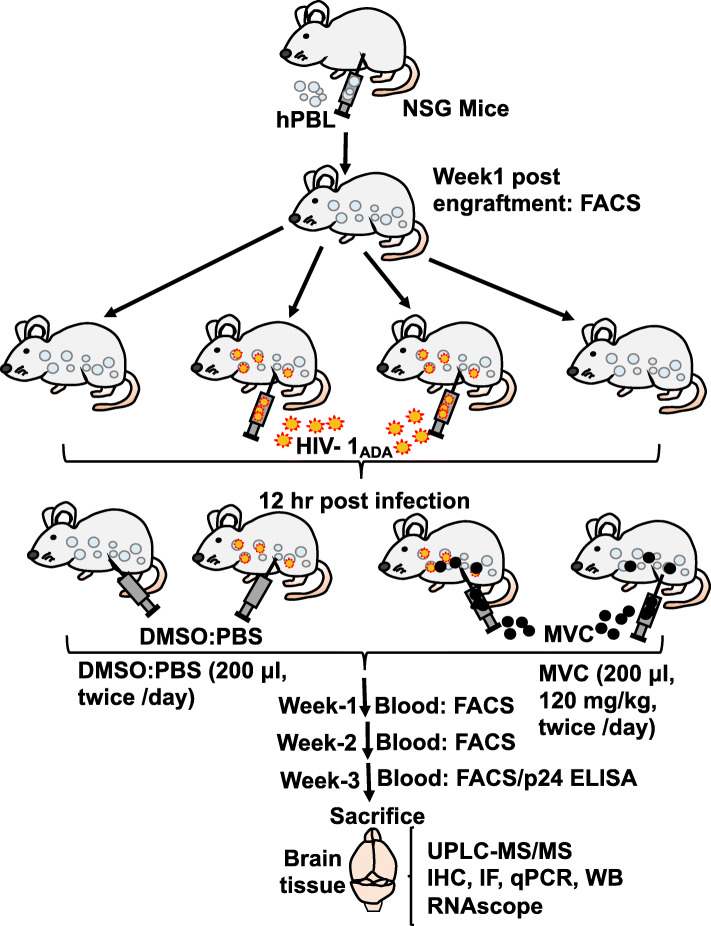
Schematic representation of animals’ engraftment, infection, treatment, samples collection, and analyses. Abbreviations: NSG: NOD/*scid*–IL-2Rγ_c_^*null*^; hPBL: human peripheral blood lymphocytes; FACS: fluorescence activated cell sorting; h: hours; MVC: maraviroc; DMSO: Dimethyl sulfoxide; PBS: phosphate-buffered saline; UPLC-MS/MS: ultraperformance liquid chromatography-tandem mass spectrometry; IHC: immunohistochemistry; IF: immunofluorescence; qPCR: quantitative (real-time) polymerase chain reaction; ELISA: enzyme-linked immunosorbent assay

Four-week-old NOD/*scid*–IL-2Rγ_c_^*null*^ (NSG) mice were purchased from the Jackson Laboratory (Bar Harbor, ME), maintained in sterile microisolator cages under pathogen-free conditions in accordance with the University of Nebraska Medical Center (UNMC) and National Institutes of Health (NIH) ethical guidelines for the care of laboratory animals, and bred at the UNMC animal facility to expand the colony. This study was performed under a protocol approved by the UNMC Institutional Animal Care and Use Committee. Human peripheral blood lymphocytes (PBL) were obtained by countercurrent centrifugal elutriation of leukopheresis packs from HIV-1, 2, and hepatitis B seronegative donors, as previously described [34]. Mice (4 to 6 weeks old males) were engrafted by intra-peritoneal (i.p.) injection of human PBL (30 × 10^6^ cells/mouse). One week after PBL injection, levels of human CD45+ cells in each animal’s blood were quantified by fluorescence activated cell sorting (FACS) to confirm engraftment. Engrafted animals were randomly assigned into 4 groups (11 to 15 mice per group): non-treated and non-infected mice (PBS); non-infected animals treated with MVC (MVC); untreated and HIV-infected mice (HIV), HIV-infected mice treated with MVC (HIV + MVC). For infection, a single dose of 10^4^ tissue culture infectious doses-50 (100 μl) of HIV-1_ADA_ (a CCR5-tropic viral strain) was injected (i.p.) into animals. Controls were mock-infected by i.p. injection of phosphate-buffered saline (PBS, 100 μl). Animals’ blood samples were collected and analyzed at week-1, week-2, and week-3 post infection (p.i.). Animals were sacrificed at week-3 p.i. and tissue samples harvested and analyzed (Fig. 1).

### Processing of brain tissues

Immediately following animals’ sacrifice, each brain lobe was cut (at the midbrain region) into two equal parts (coronal plane), for a total of 4 equal parts. One half lobe was embedded into paraffin (for immunohistochemistry), one half lobe was embedded into optimal cutting temperature (OCT) compound on dry ice and frozen at − 80 °C (for immunofluorescence analyses). The remaining two half lobes were frozen at − 80 °C and used for protein extraction and Western blot analysis, RNA extraction, and quantitative polymerase chain reaction (qPCR).

### Maraviroc (MVC) preparation

MVC (Selzentry, ViiV Healthcare) was purchased from the UNMC pharmacy in 300 mg tablets. To prepare stock concentrations of 75 mg/ml, 1 tablet was crushed using a mortar and pestle and dissolved in 4 ml of a 1:1 mixture of dimethyl sulfoxide (DMSO) and PBS. Stock solutions were stored at − 80 °C in 600 μl aliquots and used within 2 days of preparation. For animal injections, 500 μl stock solution was further diluted in 2 ml PBS for a working concentration of 18.75 mg/ml. This solution was passed through a 0.45 μm syringe filter to remove undissolvable drug excipients. Each MVC-treated mouse was injected (i.p.) with MVC (120 mg/kg, 200 μl) in a DMSO:PBS solution, twice/day, beginning at 12 h p.i. (Fig. 1). This dose and treatment schedule was based on recommended human MVC doses of 300–600 mg twice/day and the consensus human-mouse interspecies allometric scaling factor of 12.6 [35, 36]. Untreated animals were injected (i.p.) with 200 μl of a DMSO:PBS solution that had similar amounts of DMSO as the final MVC solution used for treatment.

### Antibodies

Antibodies to human CD45 (CD45-PE-Cy7, catalog [cat] #304016), CD8 (CD8-APC, cat #344722), and CD3 (CD3-Pacific blue, cat #300330) were purchased from Biolegend (San Diego, CA). Antibodies to human CD4 (CD4-FITC, cat #555346) were from BD Biosciences (San Jose, CA); human leukocyte antigen (HLA)-DR (cat #NB100-77855SS), and Aβ1–42 (cat #NBP2-13075SS) from Novus Biologicals (Centennial, CO). Antibodies for microtubule-associated protein-2 (MAP 2, cat #ab32454), neurofilament-L (NFL, cat #ab223343), NeuN (cat #ab177487), claudin-5 (cat #ab15106), zonula occludens (ZO)-1 (cat #61–7300), *β*-actin (cat #ab8226), LRP1, (cat #ab92544), RAGE (cat #ab216329), phospho(p)-Tau (Ser396) (cat #ab32057), pTau (Ser199) (cat #ab4749), pTau (Thr231) (cat #ab151559), and pTau (Thr205) (cat #ab254410) were from Abcam (Cambridge, MA). Antibodies for ZO-2 (cat #71–1400) were from Invitrogen (Carlsbad, CA); Tau (cat #46687S) and p-Tau (Thr181) (cat #12885S) from Cell signaling Technologies (Danvers, MA).

### FACS analysis

Human (hCD4+, hCD8+, hCD3+, and hCD45+) cells in animals’ blood were quantified by FACS. Briefly, blood (200 μl) collected in ethylenediaminetetraacetic acid (EDTA)-tubes were centrifuged (543×g, 8 min at 4 °C) and plasma was collected and cryopreserved. Cell pellets were resuspended in 50 μl FACS buffer (PBS containing 2% fetal bovine serum) and transferred into 5 ml polypropylene round-bottom tubes (BD Falcon, Franklin Lakes, NJ). An antibody cocktail [20 μl containing the following fluorochrome-conjugated human monoclonal antibodies: CD45-PE-Cy7, CD8-APC, CD3-Pacific blue, and CD4-FITC] was added to each sample, mixed, and incubated for 1 h on ice in the dark. One ml red blood cells lysis buffer (Roche) was then added to each sample. Samples were incubated for 5 min at room temperature (RT) and were centrifuged (377×g, 5 min at 4 °C). Cell pellets were washed two to four times in FACS buffer (2 ml), resuspended in 0.5 ml PBS containing 2% paraformaldehyde, and analyzed using BD LSRII and FACSDiva 8.0 (BD Biosciences).

### HIV p24 ELISA

Immediately following animal sacrifice, blood was collected by cardiac puncture into EDTA tubes, and plasma was obtained by centrifugation (543×g, 8 min at 4 °C) and cryopreserved. HIV-1 p24 antigen levels in each plasma sample (100 μl) were quantified using Quantikine HIV-1 group-specific antigen (Gag) p24 immunoassay kit (R&D Systems, Minneapolis, MN) per the manufacturer’s protocol; with optical density readings at 450 nm and wavelength corrections at 540 nm, using a SpectraMax M5 (Molecular Devices, San Jose, CA). Standard curves from HIV-1 Gag p24 antigen standards were used to quantify each sample’s p24 antigen levels.

### Human monocyte-derived macrophage (MDM) culture

Monocytes were obtained by countercurrent centrifugal elutriation of leukopheresis packs from HIV-1, − 2 and hepatitis-B seronegative donors, and MDM obtained as previously described [34, 37]. Briefly, freshly elutriated monocytes (2 million cells per well in 6-well plates) were differentiated into MDM by culture for 7 days in Dulbecco’s Modified Eagle’s Medium (DMEM, Sigma, St. Louis, MO) supplemented with 10% heat-inactivated pooled human serum, 1% glutamine, 50 μg/ml gentamicin, 10 μg/ml ciprofloxacin (Sigma), 1000 U/ml highly purified recombinant human macrophage colony stimulating factor. MDM were cultured as we previously described [34, 37] and all reagents were prescreened for endotoxin (< 10 pg/ml, Associates of Cape Cod, Woods Hole, MA) and mycoplasma contamination (Gen-probe II, Gen-probe, San Diego, CA).

### MDM HIV-1 infection and Aβ treatment

For infection, MDM were cultured in media containing HIV-1 (multiplicity of infection: 0.01) for 4 h, washed 3 times with serum-free media and cultured for 24 h. MDM Aβ uptake was performed as previously described [38]. Briefly, infected and non-infected MDM were cultured for 1 h in media containing human Aβ (amino acid 1–42) (Aβ-42) peptide (Invitrogen) dissolved in DMSO, at 10 μM as previously reported [38], washed 3 times with serum-free media, and cultured again for 24 h with or without MVC (2.5 or 5 μM). Controls included MDM treated with DMSO (vehicle). Culture supernatants were collected at 24 h and any cell debris removed by centrifugation (1000 x g for 10 min at 4 °C). Cells were harvested by trypsinization, washed three times with PBS, and lysed using CelLytic™ M reagent (Sigma). Cell lysates and culture supernatants were used for Aβ-42 ELISA.

### Human brain microvascular endothelial cells (HBMEC) culture

Primary HBMEC were isolated from brain tissue obtained during surgical removal of epileptogenic cerebral cortex in adult patients, under an Institutional Review Board-approved protocol as previously described [37, 39]. Routine evaluation by immunostaining for von-Willebrand factor, *Ulex europaeus* lectin and CD31 showed that cells were > 99% pure. Freshly isolated cells were cultured in collagen-coated flasks or 6-well culture plates using DMEM/F12 (Life Technologies, Grand Island, NY, USA) containing 10% fetal bovine serum (Atlanta Biologicals, Flowery Branch, GA) supplemented with 10 mM L-glutamine (Life Technologies), 1% heparin (Thermo Fisher Scientific, Pittsburgh, PA), 1% endothelial cell growth supplement (BD Bioscience, San Jose, CA), 1% penicillin-streptomycin (Life Technologies), and 1% fungizone (MP Biomedicals, Solon, OH). Cells at passage 2 to 4 were used in this study.

### HBMEC Aβ treatment

Confluent HBMEC plated on collagen-coated six-well plates were treated with human Aβ-42 peptide (10 μM), with or without MVC (5 μM) for 48 h, and LRP1 and RAGE levels in endothelial cells lysates quantified by immunoblotting. In separate experiments, HBMEC were cultured to confluence on collagen-treated tissue culture inserts (0.4-μm pore size; Corning, Lowell, MA) as we previously described [2, 40]. Human Aβ-42 peptide (10 μM) was added to the upper chamber of the transwell system in the presence or absence of MVC (2.5 or 5 μM) and/or high affinity antagonists for LRP1 (500 nM, Kerafast, Boston, MA) and RAGE (200 nM, Tocris, Minneapolis, MN) (30 min pre-treatment). These inhibitors concentrations were selected based on previously published studies [41] and manufacturers’ data showing that these antagonists concentrations blocked secretase activity and Aβ binding to LRP1 and RAGE without causing cellular toxicity. Controls included HBMEC treated with DMSO (vehicle). After 24 h culture, media in the transwell lower chamber were collected and any cell debris removed by centrifugation (1000 x g for 10 min at 4 °C). HBMEC in the transwell upper chamber were harvested by trypsinization, washed three times with PBS, and lysed. Cell lysates and culture supernatants were used for Aβ-42 ELISA.

### Amyloid-β ELISA

Levels of Aβ-42 in animals plasma samples (100 μl) were quantified using the mouse Aβ (1–42) ELISA kit (Novus Biologicals, Centennial, CO); Aβ-42 levels in human MDM and HBMEC culture supernatants and cell lysates (100 μl) were quantified using the human Aβ (1–42) Quantikine ELISA kit (R&D Systems), according to the manufacturers’ protocols. Standard curves from mouse and human Aβ (1–42) reference standards (provided with each kit) were used respectively to quantify Aβ-42 levels in plasma, MDM, and HBMEC samples.

### Immunohistochemistry

Following animal sacrifice, brain tissues (half of a brain lobe including the frontal cortex) were rinsed with PBS, fixed in 4% paraformaldehyde (overnight at 4 °C), kept in 70% ethanol for 24 h and paraffin-embedded using a Shandon Citadel 1000 tissue processor (ThermoFisher Scientific, Waltham, MA). Paraffin blocks were stored at RT and cut into 5 μm sections using a Leica RM2235 microtome (Leica Biosystems, Buffalo Grove, IL). Tissue sections were incubated for 20 to 30 s in a water bath (38 °C), mounted on Superfrost Plus microscope slides (Fisher Scientific) and dried at RT overnight. For antigen retrieval, slides were dried for 1 h at 60 °C in a standard incubator (Lab-Line 403, ThermoFisher), cooled at RT for 10 min, placed in a tray containing 300 ml of Trilogy solution (Cell Marque, Rocklin, CA) and incubated for 15 min under high pressure using a pressure cooker (Cuisinart CPC-600) containing 700 ml water as well as a 2nd tray containing 300 ml Trilogy. Following the 15 min high-pressure incubation, slides were transferred to the 2nd Trilogy tray inside the cooker, gently agitated, incubated for 5 min, and washed 5 times with deionized water, with each wash consisting of 3 min incubation in deionized water at RT. Slides were then transferred to a tray containing 300 ml PBS with 0.1% Tween-20 (PBST), incubated for 5 min at RT and for 30 min (RT) in PBST containing 10% normal goat serum (Vector, Burlingame, CA) to block non-specific sites. Slides were then incubated overnight (4 °C) with primary antibodies in PBST at the following dilutions: HLA-DR (1:100), claudin-5 (1:250), NeuN (1:100), MAP 2 (1:3500), Aβ1–42 (1:500), Tau (1:500), and phospho-Tau (1:100). Control antibodies included isotope-matched IgG.

Following incubation with primary antibodies, slides were washed 3 times with PBST at RT (5 min for each wash), incubated for 1 h at RT with polymer-based horseradish peroxidase (HRP)-conjugated EnVision mouse or rabbit secondary antibodies (Dako, Carpinteria, CA) and washed 3 times with PBST. Slides were developed with 3,3′-diaminobenzidine (DAB; Dako), counter-stained with hematoxylin (for 30 s), washed three times with deionized water, dipped for 10 s in ammonia (0.037 mol/l) and rinsed three times with deionized water. Slides were dehydrated by sequential incubation (5 min, RT) in ethanol: in 80% (once), 95% (once), and 100% (twice) ethanol. Dehydrated slides were washed (5 min, RT) twice in xylene, air dried for 2 min, and mounted using Cytoseal-60 (ThermoFisher). A coverslip was then placed over each tissue, avoiding the formation of air bubbles.

Images were captured using a Nikon Eclipse E800 microscope, Infinity-1(IFN 1-5C) camera (Luminera, Ontario, Canada) and the Infinity Analyze software. Quantitative analysis of HLA-DR+ cells was performed using the computer-assisted image analysis of the MetaMorph software (Molecular Devices, San Jose, CA). For each mouse, ten fields-of-view (FOV) were analyzed and normalized to surface area to estimate the number of HLA-DR+ cells per μm^2^ of FOV. Semi-quantitative analysis of claudin-5, NeuN, MAP 2, Aβ, Tau, and phospho-Tau expression (staining intensity and surface area occupied by immunostaining) was performed using the MetaMorph software. For each mouse, ten FOV were analyzed, the staining intensity was normalized to surface area (μm^2^) and averaged to estimate the protein expression (μm^2^ FOV). Coronal sections from the somatosensory regions of the cerebral cortex were used for all histology, except for Tau and pTau where we used coronal sections from the hippocampus fimbria (Supplemental Fig. 1) because pTau were mostly concentrated in this region.

### RNAscope assay

This assay was performed using RNAscope® 2.5 Assay kit (ACD Biotech, Newark, CA) according to the manufacturer’s protocol. Briefly, 5 μm paraffin-embedded brain tissue (frontal cortex) sections were mounted onto slides, air dried overnight, and baked for 1 h at 60 °C in a standard incubator. Baked tissues were immediately deparaffinized by washing (5 min incubation at RT) three times with xylene, incubated for 1 min in 100% ethanol, and dried at RT for 5 min. Tissues were incubated for 10 min (RT) in hydrogen peroxide, 15 min in RNAscope target retrieval buffer heated to 99 °C, rinsed in distilled water, and incubated for 3 min (RT) in 100% ethanol. Slides were then air-dried and kept on an HybEZ rack (ACD Biotech) placed in a humidified chamber. RNAscope protease plus solution was added to tissues and slides incubated for 30 min at 40 °C in the HybEZ oven (ACD Biotech), washed in distilled water, treated with 78-ZZ HIV RNA specific probe (ACD Biotech), incubated again at 40 °C for 2 h, and washed three times with the wash buffer provided with the kit. Detection of RNA-specific probes hybridized to target viral RNA was done by sequential hybridization with HRP-labeled probes and chromogenic detection using the DAB system, with hematoxylin counterstaining and ethanol dehydration. Dehydrated slides were washed (5 min, RT) twice in xylene, air dried for 2 min, and mounted using Cytoseal-60 and a coverslip. Images were captured using a Nikon Eclipse E800 microscope, Infinity-1(IFN 1-5C) camera (Luminera, Ontario, Canada) and the Infinity Analyze software. Quantitative analysis of RNA copies was performed using a computer-assisted image analysis of the MetaMorph software. The total number of viral RNA in each cluster was calculated by area normalization. For each mouse, ten FOV were analyzed and averaged to estimate HIV RNA copy number per μm^2^ of FOV.

### Immunofluorescence

OCT-embedded brain tissue sections (10 μm) were mounted onto Superfrost Plus slides, fixed in 4% formaldehyde (20 min, RT), dried (10 min), washed in PBS (5 min, RT), and incubated (1 h, RT) in PBS containing 3% bovine serum albumin and 0.1% triton X100 to block non-specific bindings. Slides were then incubated overnight (4 °C) with primary antibodies in blocking solution, at the following dilutions: NFL (1:4000), ZO-1 (1:1000), and ZO-2 (1:500). Following incubation with primary antibodies, slides were washed (5 min, RT) three times with PBS, incubated (1 h in the dark at RT) with secondary antibodies conjugated to Alexa Fluor-488 (diluted 1:5000 in blocking solution), washed five times with PBS, and mounted in Prolong Gold anti-fade reagent containing DAPI (Molecular Probes, Grand Island, NY). Images were captured using an Eclipse TE20000-U fluorescent microscope (Nikon, Melville, NY) and Infinity 3-6urfm monochrome camera (Luminera). Semi-quantitative analysis of NFL, ZO-1, and ZO-2 expression was performed using computer-assisted image analysis of the MetaMorph software. For each mouse, ten FOV were analyzed, the staining intensity was normalized to surface area (μm^2^) and averaged to estimate the protein expression (μm^2^ FOV).

### Ultraperformance liquid chromatography-tandem mass spectrometry (UPLC-MS/MS)

MVC levels in animals’ plasma and brain tissues (cerebrum) were quantified by UPLC-MS/MS as previously described [42]. For plasma, 50 μl of each plasma sample was added to 10 μl of a 1 μg/ml indinavir free base as internal standard (IS) and 1 ml of ice-cold MS-grade acetonitrile. For brain tissue, 100 mg of each tissue sample was homogenized in four volumes of MS-grade water; 1 ml ice-cold acetonitrile containing 10 μl of IS was then added. Both plasma and brain samples were vortexed for 3 min and centrifuged (16,000×g for 10 min, 4 °C). One ml of supernatant was evaporated to dryness under vacuum and dried samples reconstituted in 100 μl of 50% MS-grade methanol in water. Samples were centrifuged (16,000×g for 10 min, 4 °C) and 40 μl of supernatant was used for analysis.

Chromatographic separation was performed using a Waters ACQUITY UPLC (Milford, MA) system coupled with a Sciex QTRAP 4500 triple quadrupole linear ion trap hybrid mass spectrometer, with an electrospray ionization source (Applied Biosystems /MDS Sciex, Foster City, CA). For separation, an ACQUITY BEH Shield RP18 column (1.7 μm, 2.1 × 100 mm) equipped with an ACQUITY Vanguard BEH Shield precolumn (1.7 μm, 2.1 × 5 mm) was employed, using a stepwise gradient of 7.5 mM ammonium acetate, pH 5 for mobile phase A and acetonitrile for mobile phase B. The gradient was held at 70% mobile phase A for 3 min, decreased to 40% mobile phase A over 90 s and held for 30 s, decreased to 5% mobile phase A over 30 s and held for 30 s, increased to 70% mobile phase A over 15 s and held for 105 s prior to next sample injection at a flow rate of 0.25 ml/min. The injection volume for each sample was 10 μl. Detection was achieved in the positive ionization mode using the following transitions: m/z MVC 514/280; m/z indinavir 614/421. Calibration standards consisted of 0.2 to 2000 ng/ml MVC with 100 ng/ml indinavir for both plasma and brain homogenates and the ratio of analyte to IS peak area was used for quantitation of unknowns.

### RNA isolation and real-time PCR

Total RNA was extracted from brain tissues using Trizol reagent (Life Technologies-Ambion, Austin, TX) according to the manufacturer’s protocol. RNA yield and quality were checked using a NanoDrop spectrophotometer (NanoDrop Technologies, Wilmington, DE) and for all samples, absorbance ratios of 260/280 were ≥ 2. Reverse transcription was performed using Verso cDNA synthesis kit (ThermoFisher); 1 μg RNA in 11 μl of nuclease-free water was mixed with 4 μl of 5X cDNA synthesis buffer, 2 μl dNTP mix, 1 μl random hexamers, 1 μl reverse transcriptase enhancer and 1 μl Verso enzyme mix. Amplification conditions were: 1 cycle of 42 °C for 30 min, followed by 95 °C for 2 min.

Quantitative real-time PCR was performed using the 384-well block of a LightCycler® 480 II (Roche) Real-Time PCR System. For each reaction, 500 ng of cDNA in 5 μl nuclease-free water was mixed with 3 μl PCR grade water, 10 μl of 2X LightCycler® 480 Probe master mix, and 1 μl of 20X TaqMan primer-probe mix. Cycling conditions were as follows: 95 °C, 5 min with a ramp rate of 4.8 °C/s; followed by 45 cycles of 95 °C, 10 s, 4.8 °C/s; 60 °C, 15 s, 2.5 °C/s; and 72 °C, 1 s, 4.8 °C/s; and hold at 40 °C, 10 s, 2 °C/s. Standard curves were generated from ACH-2 cells (an HIV-1 latent T-cell clone containing one integrated copy of proviral DNA per cell), and qPCR was used to quantify HIV-1 long-terminal repeat (LTR), polymerase (pol), transactivator of transcription (tat), and gag copy numbers in each sample. Results were further normalized to levels of human CD45+ cells in each brain tissue sample. MAP-2, NeuN, gamma-secretase activating protein (GSAP) and NFL mRNA levels were quantified using the delta-CT method as instructed in the Lightcycle 480 software manual and normalized to the sample’s GAPDH levels. All primers were obtained from Applied Biosystems, and primers’ IDs were as follows: LTR (AIWR3QG), pol (AIY9Z2W), tat (AIX01W0), gag (AIo1X84), CD45 (Hs04189704), MAP-2 (Mm00485231), NFL (Mm01315666_m1), NeuN (Mm01248771_m1), GSAP (Mm00615236_m1) and GAPDH (Mm99999915_g1).

### Western blot analysis

Each brain tissue sample (5 mg) was transferred into a tube containing 500 μl of ice-cold tissue lysis buffer (50 mM Tris-HCl, 150 mM NaCl, 0.25% SDS, 0.25% Sodium Deoxycholate, 1 mM EDTA) and 5 μl of 100X protease and phosphatase inhibitor cocktail, placed on ice and homogenized using a motor and pestle. Homogenized samples were incubated for 30 min on ice, with intermittent vortex (every 5 to 6 min) for 10 s. Samples were centrifuged for 10 min at 18000×g, 4 °C; and each supernatant transferred into a fresh pre-chilled tube and stored at − 80 °C until use. Total protein levels in each sample were quantified using the bicinchoninic acid assay (ThermoFisher), and 30 μg protein analyzed by sodium dodecyl sulfate-polyacrylamide gel electrophoresis as previously described [34, 43] using monoclonal antibodies to claudin-5, ZO-1, ZO-2, MAP 2, NeuN, NFL, LRP1, RAGE, Aβ1–42, Tau, pTau (Thr181), pTau (Ser396), pTau (Ser199), pTau (Thr231), pTau (Thr205), and *β*-actin (each at 1:1000 dilution). To confirm equal loading, protein expression in each sample was normalized to the sample’s *β*-actin levels, and pTau normalized to the sample’s total Tau levels. The original Western blot images are included in “Additional File-1”.

### Statistical analysis

Data were analyzed by Student’s t-test (two-tailed) or by one- or two-way analysis of variance followed by Tukey’s multiple-comparisons tests using GraphPad Prism 7.05 (GraphPad Software, La Jolla, CA, USA). Data are presented as mean ± standard deviation (SD) and the threshold of significance level was 0.05.

## Results

### MVC reduced HIV-induced immunosuppression in infected animals

**Fig. 2:**
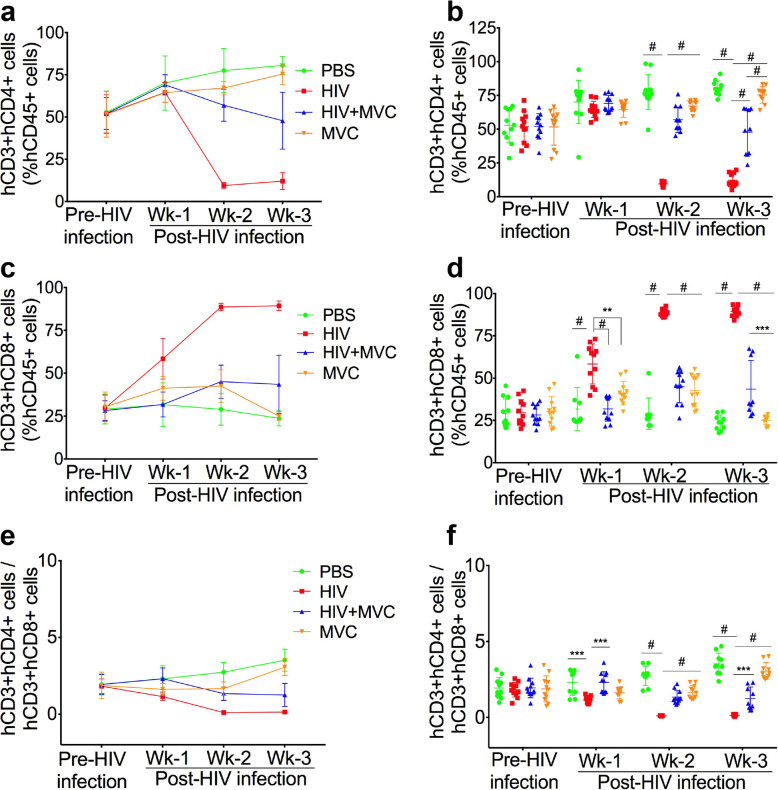
MVC prevents HIV-induced immunosuppression in infected mice. Levels of human (h)CD4+ (**a**, **b**), hCD8+ (**c**, **d**) and hCD45+ T-cells in each blood sample were quantified by FACS before (Pre) infection and at week (Wk) -1, − 2, and − 3 post-infection. Levels of hCD4+ and hCD8+ cells in each sample were normalized to the sample’s hCD45+ cells levels. **e**, **f**: hCD4+/hCD8+ T-cells ratios. The four animal groups included NSG mice engrafted with human PBL, uninfected and untreated (PBS), uninfected and treated with MVC (MVC), infected with HIV-1_ADA_ and untreated (HIV), or treated with MVC (HIV + MVC). Sample size: 9 to 11 animals in each group. ^#^*P* < 0.0001, ***P < 0.0007, **P = 0.0013. For all panels, error bars represent standard deviation (SD)

HIV infection is known to decrease CD4+ T-cells and increase CD8+ T-cells [44, 45]. To determine the effects of HIV infection and MVC treatment on the animals’ immune system, we quantified blood levels of human (hCD45+, hCD3+, hCD4+, and hCD8+) cells in animals’ blood before (pre) infection and at week-1, week-2, and week-3 p.i. The four animal groups showed no significant difference in mean hCD4+ cells pre-infection (51.65 ± 13.54 (SD) to 52.85 ± 12.54%) or at week-1 p.i. (64.5 ± 5.7 to 70 ± 16%) (Fig. 2a and b). At week-2 p.i., infected untreated animals (HIV) showed major immunosuppression compared to infected animals treated with MVC (HIV + MVC), non-infected controls (PBS), or non-infected animals treated with MVC (MVC). The mean number of hCD4+ T-cells in the HIV group was 8-fold lower than in the PBS group (*P* < 0.0001), 6-fold lower than in the HIV + MVC group (P < 0.0001), and 7-fold lower than in the MVC group (P < 0.0001) (Fig. 2b). At week-3 p.i., the mean number of hCD4+ cells in the HIV group was 6.6-fold lower than in the PBS group (*P* < 0.0001), 4-fold lower than in the HIV + MVC group (P < 0.0001), and 6.2-fold lower than in the MVC group (P < 0.0001) (Fig. 2b). Thus, compared to untreated HIV-infected animals, infected animals treated with MVC showed 6-fold higher hCD4+ T-cells at week-2 and 4-fold higher hCD4+ T- cells at week-3 p.i.

### MVC reduced HIV-induced increase in hCD8+ T-cells in infected animals

Pre-infection mean levels of hCD8+ T-cells were not significantly different between the 4 animal groups (28 ± 5.7 to 30.4 ± 8.66%) (Fig. 2c and d). However, HIV infection resulted in increased hCD8+ T-cells, which was prevented by MVC treatment. At week-1 p.i., mean hCD8+ T-cells in infected animals were 1.8-fold higher than in infected mice treated with MVC or PBS control (*P* < 0.0001), and 1.4-fold higher than hCD8+ T-cells in the MVC control group (*P* = 0.0013) (Fig. 2c and d). At week-2 p.i., mean hCD8+ T-cells in infected animals were 1.96-fold, 3-fold, and 2.1-fold higher than the mean hCD8+ T-cells in the HIV + MVC, PBS, and MVC groups, respectively (*P* < 0.0001, Fig. 2d). At week-3 p.i., mean hCD8+ T-cells in the HIV group were 2-fold, 3.76-fold, and 3.56-fold higher than mean hCD8+ T-cells in the HIV + MVC, PBS, and MVC groups, respectively (*P* < 0.0001, Fig. 2d).

### MVC increases hCD4+/hCD8+ T-cells ratios in infected animals

Pre-infection, hCD4+/hCD8+ T-cells ratios were not significantly different between the 4 animal groups (1.82 to 1.94). HIV infection resulted in decreased hCD4/hCD8 ratios, which was abrogated by MVC treatment. Compared to infected untreated mice, infected mice treated with MVC, or PBS control had 2-fold higher hCD4/hCD8 ratios at week-1 p.i. (*P* = 0.0004, Fig. 2e and f). At week-2 p.i., compared to infected untreated mice, hCD4/hCD8 ratios were 12.5-fold, 25.6-fold, and 15.5-fold higher in HIV + MVC, PBS, and MVC animal groups, respectively (*P* < 0.0001, Fig. 2f). At week-3 p.i., compared to infected untreated mice, hCD4/hCD8 ratios were 9.2-fold, 25.78-fold, and 22.4-fold higher in HIV + MVC (*P* = 0.0007), PBS, and MVC groups (*P* < 0.0001), respectively (Fig. 2f).

### MVC enters the CNS and decreases systemic and brain viremia in infected animals

We performed UPLC-MS/MS quantification of MVC in plasma and brain tissues of mice treated with MVC (at week-3) as detailed in the Methods section. The mean MVC plasma levels were 117.5 ± 120.6 ng/ml (range: 7.23 to 294 ng/ml), and the mean MVC levels in brain tissues were 216.6 ± 234 ng/g (range: 81 to 685 ng/g). There was a positive correlation between plasma and brain MVC levels (Pearson r = 0.638), but it did not reach statistical significance (*P* = 0.09).

**Fig. 3:**
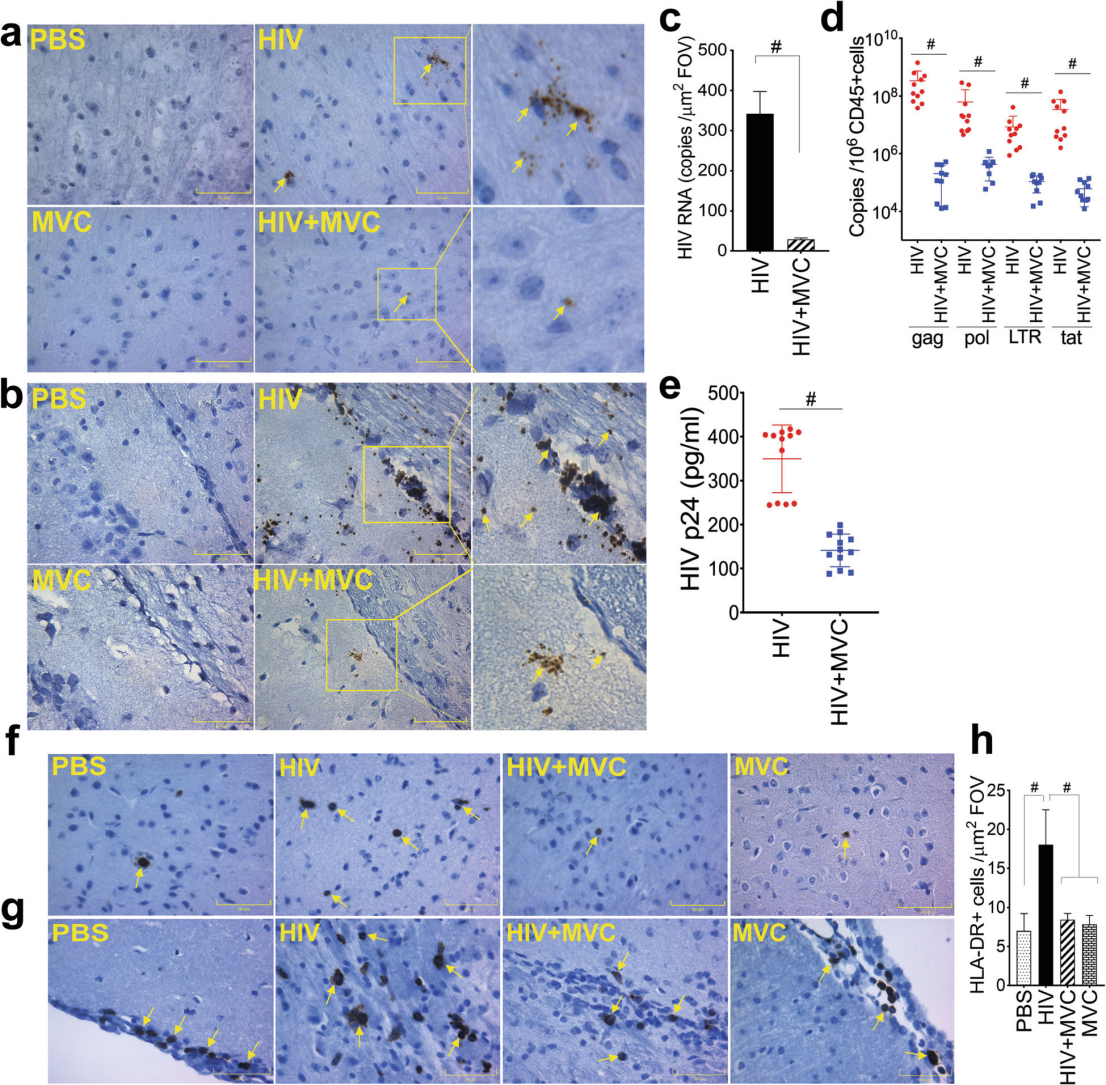
MVC decreased viremia and abrogated HIV-induced cellular infiltration in the brain of infected animals. Brain HIV RNA copy (yellow arrows) numbers were quantified by RNAscope and, for each experimental group, representative images from the somatosensory cortex (**a**) and meningeal / somatosensory area layer 1 (**b**) are shown. **c**: Metamorph software was used to quantify HIV RNA copies numbers in all samples. For each animal brain sample, 10 random fields-of-view (FOV) were analyzed (5 FOV from the somatosensory cortex and 5 FOV from the meningeal / somatosensory area layer 1). **d**: Levels of HIV-1 gag, pol, LTR, and tat genes in brain tissues were quantified by qPCR and normalized to samples’ hCD45+ cells levels. **e**: HIV-1 p24 antigen levels in plasma samples were quantified by ELISA. **f, g**: HLA-DR expression in brain tissues was analyzed by immunohistochemistry and, for each experimental group, representative images from the somatosensory cortex (**f**) and meningeal / somatosensory area layer 1 (**g**) are shown. **h**: Metamorph was used to quantify HLA-DR+ cells in all brain samples and for each sample, 10 random FOV (5 FOV from the somatosensory cortex and 5 FOV from the meningeal / somatosensory area layer 1) were analyzed. For panels **a, b, f,** and **g**, images were at 40X. The four animal groups included PBS, HIV, HIV + MVC, and MVC; 9 to 12 animals in each group. #*P* < 0.0001. Error bars represent SD

RNAscope quantification of HIV in brain tissues showed high viral copy numbers in the brains of infected animals and MVC treatment decreased the brain viral loads by 11.75-fold (Fig. 3a-c, *P* < 0.0001, degree of freedom (df) = 14, F = 258.6). qPCR quantification of viral genes also showed high levels of HIV-1 gag, pol, LTR and tat in brain tissues of infected animals, and MVC treatment significantly decreased the levels of each of these viral genes (Fig. 3d, P < 0.0001). Mean HIV-1 p24 antigen levels in the plasma of infected mice were 350 ± 79 pg/ml, and MVC treatment decreased plasma viral p24 levels by 2.5-fold (mean p24: 141 ± 37 pg/ml) (Fig. 3e, P < 0.0001, df = 22). Two-tailed t-tests were used for Fig. 3c-e.

### MVC blocked HIV-induced leukocyte infiltration into the brain

Quantification of human (HLA-DR+) cells in animals’ brain tissues showed significantly higher cellular infiltration in the brain of HIV-infected animals. Compared to non-infected controls (PBS or MVC groups), mean HLA-DR+ cells in the brain of HIV-infected animals were 2.3 to 2.58-fold higher (Fig. 3f-h). MVC treatment (HIV + MVC) decreased HLA-DR+ cells in the brain of infected animals by 2.13-fold compared to infected and non-treated animals (Fig. 3h, *P* < 0.0001).

### MVC prevented HIV-induced BBB alterations

**Fig. 4:**
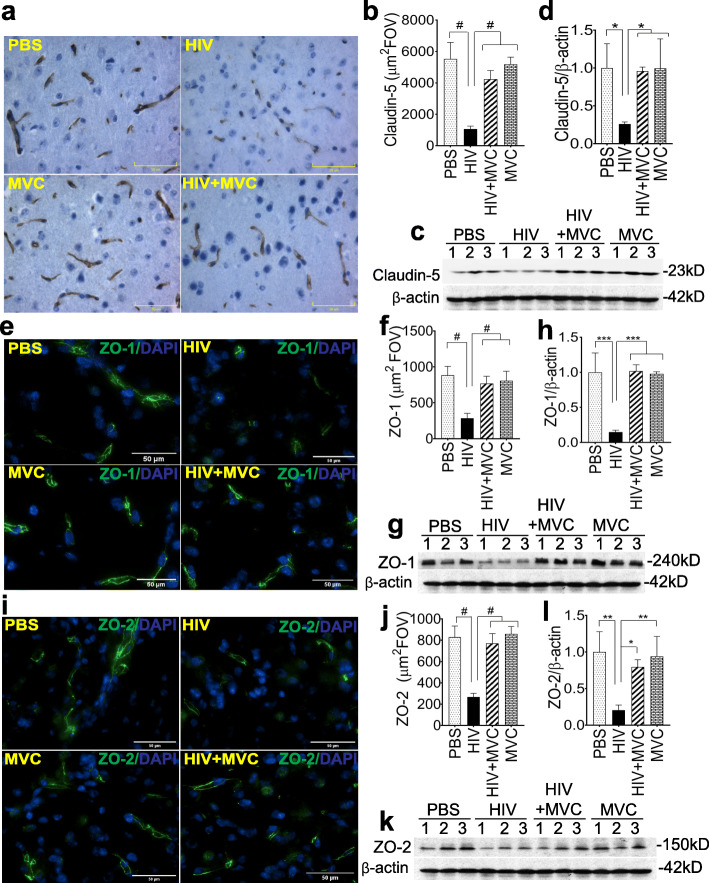
MVC abrogated HIV-induced BBB alterations. Expression of brain endothelial tight junction proteins claudin-5 (**a-d**), ZO-1 (**e-h**), and ZO-2 (**i-l**) were analyzed by immunohistochemistry (**a, b**) or immunofluorescence (**e, f, i, j**) with DAPI (blue) for nuclear counterstaining. Metamorph software was used to quantify claudin-5 (**b**), ZO-1 (**f**), and ZO-2 (**j**) levels in all samples; for each sample, 10 random FOV (from the somatosensory cortex) were analyzed. Western blot analyses (**c**, **g**, **k**) and densitometry quantification normalized to sample’s β-actin levels (**d**, **h**, **l**) were also used to quantify claudin-5 (**c, d**), ZO-1 (**g, h**), and ZO-2 (**k, l**) levels in samples. For panels **a**, **e**, and **i**, images were at 40X. The four animal groups included PBS, HIV, HIV + MVC, and MVC; 9 to 11 animals in each group. ^#^P < 0.0001, ***P < 0.0005, **P < 0.006, *P < 0.05. Error bars represent SD

To assess the effects of HIV and MVC treatment on the BBB in vivo, we analyzed the expression of endothelial tight junction (TJ) proteins claudin-5 (Fig. 4a-d), ZO-1 (Fig. 4e-h), and ZO-2 (Fig. 4i-l) in animals’ brain tissues. Immunohistochemistry and immunofluorescence analyses showed a decreased expression of claudin-5 (Fig. 4a and b), ZO-1 (Fig. 4e and f), and ZO-2 (Fig. 4i and j) in brain tissues of infected animals, and MVC prevented HIV-induced downregulation of these TJ proteins. Metamorph quantification of TJ proteins expression in all animals (9 to 11 mice in each group) showed that HIV-1 infection decreased claudin-5 expression by 5.2-fold (Fig. 4b, P < 0.0001) and decreased ZO-1 (Fig. 4f) and ZO-2 (Fig. 4j) expression by 3-fold (P < 0.0001) compared to PBS control. Compared to infected and non-treated animals, expression of claudin-5, ZO-1, and ZO-2 in the HIV + MVC group were increased, respectively, by 4-fold (Fig. 4b), 2.7-fold (Fig. 4f), and 2.88-fold (Fig. 4j) (*P* < 0.0001). These results were confirmed by Western blot analyses (Fig. 4c, g and k) and densitometry quantification (Fig. 4d, h and l). Data normalized to samples’ actin levels showed that compared to the control PBS group, HIV-1 infection decreased the expression of claudin-5, ZO-1, and ZO-2, respectively, by 3.8-fold (Fig. 4d, *P* = 0.03), 6.7-fold (Fig. 4h, *P* = 0.0005) and 4.85-fold (Fig. 4l, *P* = 0.006). Compared to the HIV group, expression of claudin-5, ZO-1, and ZO-2 in the HIV + MVC group was increased, respectively, by 3.6-fold (Fig. 4d, *P* = 0.04), 6.8-fold (Fig. 4h, *P* = 0.0004) and 4.56-fold (Fig. 4l, P = 0.03).

### MVC reduced HIV-induced neuronal injury in infected animals

**Fig. 5:**
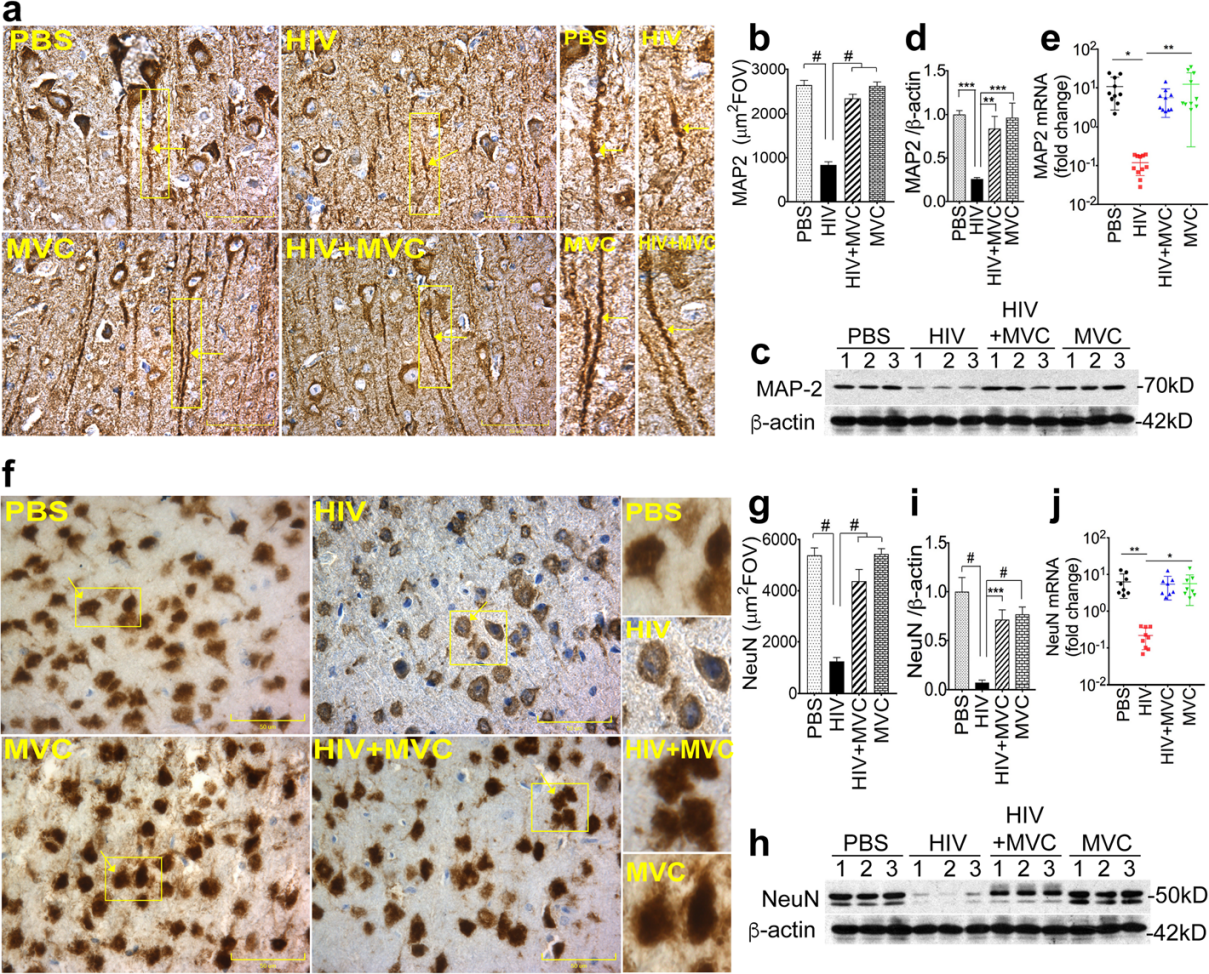
MVC prevented HIV-induced downregulation of the neuronal markers MAP 2 and NeuN. **a**: Immunohistochemistry analyses of MAP 2 expression in brain tissues (somatosensory cortex). **b**: Metamorph quantification of MAP 2 expression in all samples. MAP 2 levels in brain tissues were also quantified by Western blot (**c**) followed by densitometry quantification normalized to sample’s β-actin levels (**d**). **f**: immunohistochemistry analyses of NeuN expression in brain tissues (somatosensory cortex). **g**: Metamorph quantification of NeuN in all samples. NeuN levels in brain tissues were also quantified by Western blot (**h**) followed by densitometry quantification normalized to sample’s β-actin levels (**i**). MAP 2 (**e**) and NeuN (**j**) mRNA levels in brain tissues were quantified by real-time PCR. For panels **a** and **f**, images were at 40X. For panels **b** and **g**, 10 random FOV analyzed for each sample. The four animal groups included PBS, HIV, HIV + MVC, and MVC; 9 to 11 animals in each group. ^#^P < 0.0001, ***P < 0.0003, **P < 0.004, *P < 0.015. Error bars represent SD

To determine the effect of HIV infection and MVC on neuronal processes and phenotypes, we analyzed the expression of neuronal cytoskeletal (MAP 2), nuclei (NeuN), and axonal (NF-L) markers in brain tissues. HIV infection decreased MAP 2 (Fig. 5a-e) and NeuN (Fig. 5f-j) expression, effects that were blocked by MVC treatment. Immunohistochemistry and metamorph quantification of MAP 2 and NeuN expression in brain tissues showed that compared to control animals (PBS or MVC groups), HIV-1 infection decreased MAP 2 (Fig. 5a and b) and NeuN (Fig. 5f and g) expression by 3.16-fold and 4.35-fold, respectively, and MVC treatment blocked HIV-induced downregulation of MAP 2 and NeuN. In the HIV + MVC group, MAP 2 (Fig. 5a and b) and NeuN (Fig. 5f and g) expression increased by 2.8 and 3.53-fold (*P* < 0.0001). Western blot analyses of brain tissues confirmed these findings. Compared to control animals, HIV-1 infection decreased MAP 2 (Fig. 5c and d) and NeuN (Fig. 5h and i) expression by 3.8 and 15-fold, respectively; whereas in the HIV + MVC group, MAP 2 and NeuN expression increased by 3.2-fold (Fig. 5c and d, *P* < 0.01) and 10.6-fold (Fig. 5h and i, *P* < 0.001), respectively, compared to animals in the HIV group. qPCR also showed that HIV infection decreased MAP 2 mRNA levels by 90.7-fold (Fig. 5e, *P* = 0.011) and decreased NeuN mRNA by 28.7-fold (Fig. 5j, *P* = 0.004). In infected animals treated with MVC, MAP 2 and NeuN mRNA levels were increased by 46.8-fold (Fig. 5e *P* = 0.003) and 25-fold (Fig. 5j, *P* = 0.015), respectively, compared to levels in infected untreated animals.

**Fig. 6:**
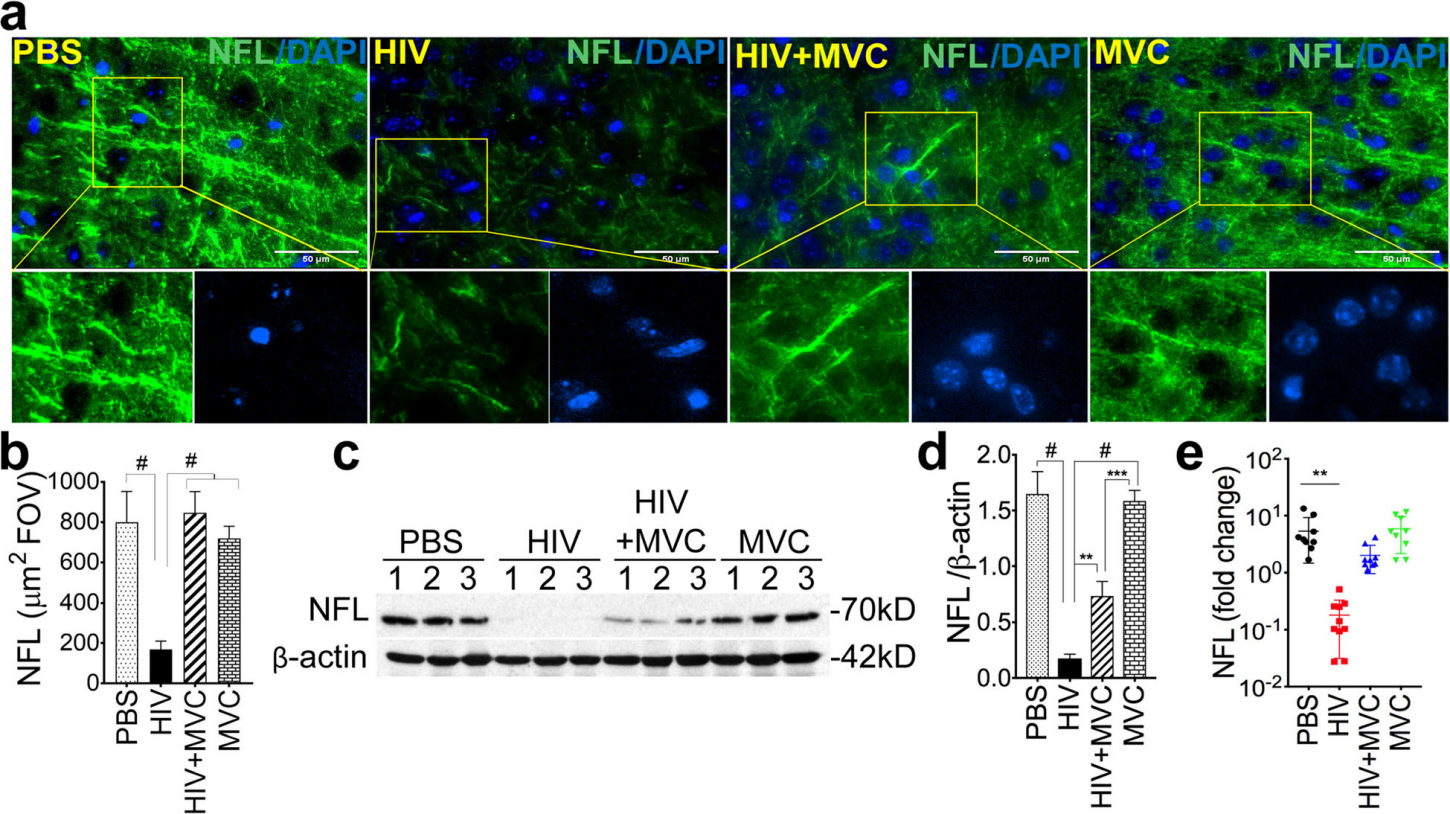
MVC prevented HIV-induced downregulation of the neurofilament-L (NFL). **a**: immunofluorescence analyses of NFL expression in brain tissues. **b**: densitometry quantification of NFL expression in all samples (somatosensory cortex, 10 random FOV analyzed for each sample). NFL levels in animals’ brain tissues were also quantified by Western blot analyses (**c**) followed by densitometry quantification normalized to sample’s β-actin levels (**d**). **e**: Real-time PCR quantification of NFL mRNA in brain tissues. For panel **a**, images were at 40X. The four animal groups included PBS, HIV, HIV + MVC and MVC; 9 to 11 animals in each group. ^#^P < 0.0001, ***P < 0.0002, **P < 0.003. Error bars represent SD

Immunofluorescence and metamorph quantification showed that compared to control (PBS and MVC) groups, HIV infection decreased NFL expression by 4.75-fold (Fig. 6a and b; *P* < 0.0001); and NFL levels in the HIV + MVC group were increased by 5-fold compared to the HIV group (Fig. 6a and b, P < 0.0001). Western blot analyses also showed that HIV infection decreased NFL expression by 9.35-fold (Fig. 6c and d, *P* < 0.0001), and MVC treatment increased NFL expression in infected animals by 4-fold compared to infected and untreated animals (Fig. 6c and d, P = 0.003). HIV infection also decreased NFL mRNA levels in brain tissues by 29.5-fold (Fig. 6e, *P* = 0.001), and NFL mRNA levels in the HIV + MVC group were 11-fold higher than levels in infected untreated animals (Fig. 6).

### HIV-1 infection increased CNS and plasma Aβ and increased CNS GSAP

**Fig. 7:**
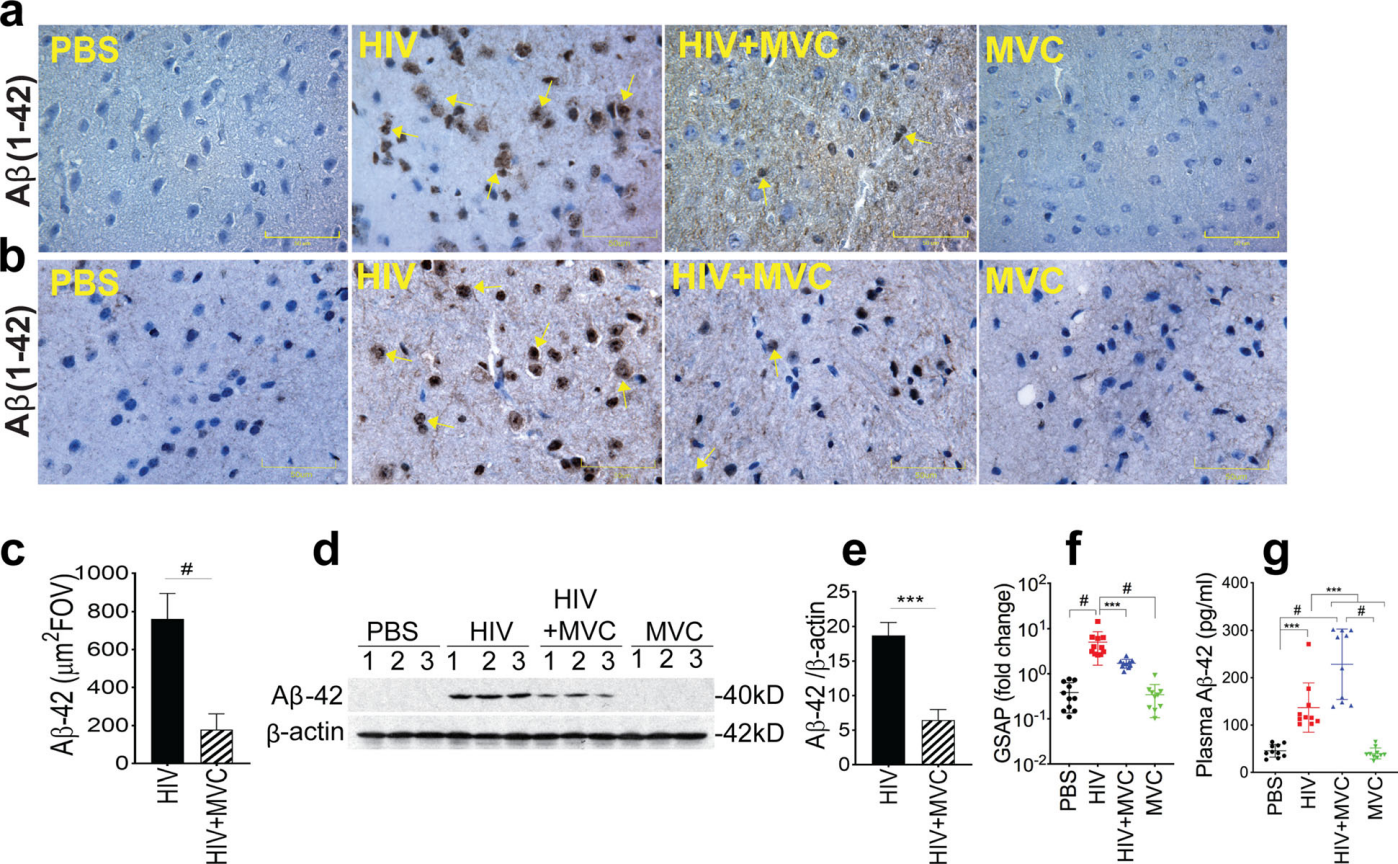
MVC abrogated HIV-induced increased formation and accumulation of CNS GSAP and amyloid-β (Aβ), but increased plasma Aβ levels. Aβ formation in the somatosensory cortex (**a**) and hypothalamus region (**b**) were quantified by immunohistochemistry using antibodies to Aβ1–42, followed by densitometry quantification of Aβ levels in all samples: 10 random FOV (5 FOV from the somatosensory cortex and 5 FOV from the hypothalamus region) analyzed for each sample (**c**). Aβ levels in brain tissues were also analyzed by Western blot (**d**) and densitometry quantification normalized to sample’s β-actin levels (**e**). Both immunohistochemistry and Western blot showed no Aβ in brain tissues of animals in the PBS or MVC groups. mRNA levels of GSAP (the enzyme that catalyzes Aβ formation) in brain tissues were quantified by real-time PCR (**f**). Plasma Aβ1–42 were quantified by ELISA (**g**). For panels **a**, and **b**, images were at 40X . The four animal groups analyzed included PBS, HIV, HIV + MVC, and MVC; 9 to 11 animals in each group. ^#^P < 0.0001, ***[(**e**) *P* = 0.0009, (**f**) *P* = 0.0008, (**g**) *P* = 0.0005]. Error bars represent SD

HAND is associated with AD-like pathology characterized by increased CNS Aβ and Tau hyperphosphorylation [17–20]. Immunohistochemistry (Fig. 7a-c) and Western blot (Fig. 7d, e) analyses of brain tissues showed no Aβ-42 in control animals, but brain tissues from HIV-infected animals showed increased formation and accumulation of Aβ-42 (Fig. 7a-e). qPCR analyses of GSAP also showed that HIV-1 infection increased GSAP mRNA in brain tissues by 13 to 15-fold compared to control animal groups (Fig. 7f, *P* < 0.0001). ELISA quantification of Aβ-42 in animals’ plasma at week-3 p.i. showed that HIV infection increased plasma Aβ-42 levels by 3-fold. Plasma Aβ-42 levels in HIV-infected animals were 137 ± 52 pg/ml compared to 45.7 ± 13.5 pg/ml in control PBS animals (*P* = 0.0005, Fig. 7g).

### MVC reduced HIV-induced GSAP and CNS Aβ formation, but increased plasma Aβ levels

qPCR showed that MVC treatment of infected animals reduced GSAP mRNA levels in brain tissues by 3-fold compared to animals in the HIV group (Fig. 7f, *P* = 0.0008). Immunohistochemistry analyses showed that compared to infected (HIV group) animals, MVC treatment (HIV + MVC group) reduced Aβ-42 levels by 4.26-fold [Fig. 7a-c, *P* < 0.0001; df = 14; F = 2.57]. Western blot analyses confirmed these findings and showed that MVC treatment of infected animals reduced Aβ-42 levels by 3-fold (Fig. 7d and e, *P* = 0.0009; df = 4, F = 21.49) compared to animals in the HIV group. Two-tailed t-tests were used for Fig. 7c and e. For animals in the PBS and MVC groups, immunohistochemistry (Fig. 7a-c) and Western blot (Fig. 7d, e) analyses showed no detectable Aβ-42 in brain tissues. Surprisingly, plasma Aβ-42 levels in HIV-infected and MVC-treated animals were 1.67-fold higher (228.6 ± 74.3 pg/ml) than in infected non-treated animals (Fig. 7g, P = 0.0005), 5-fold higher than in control PBS group, and 5.7-fold higher than in the control MVC group (40 ± 11.6 pg/ml) (Fig. 7g, *P* < 0.0001).

### HIV-1 infection increased CNS tau phosphorylation

**Fig. 8:**
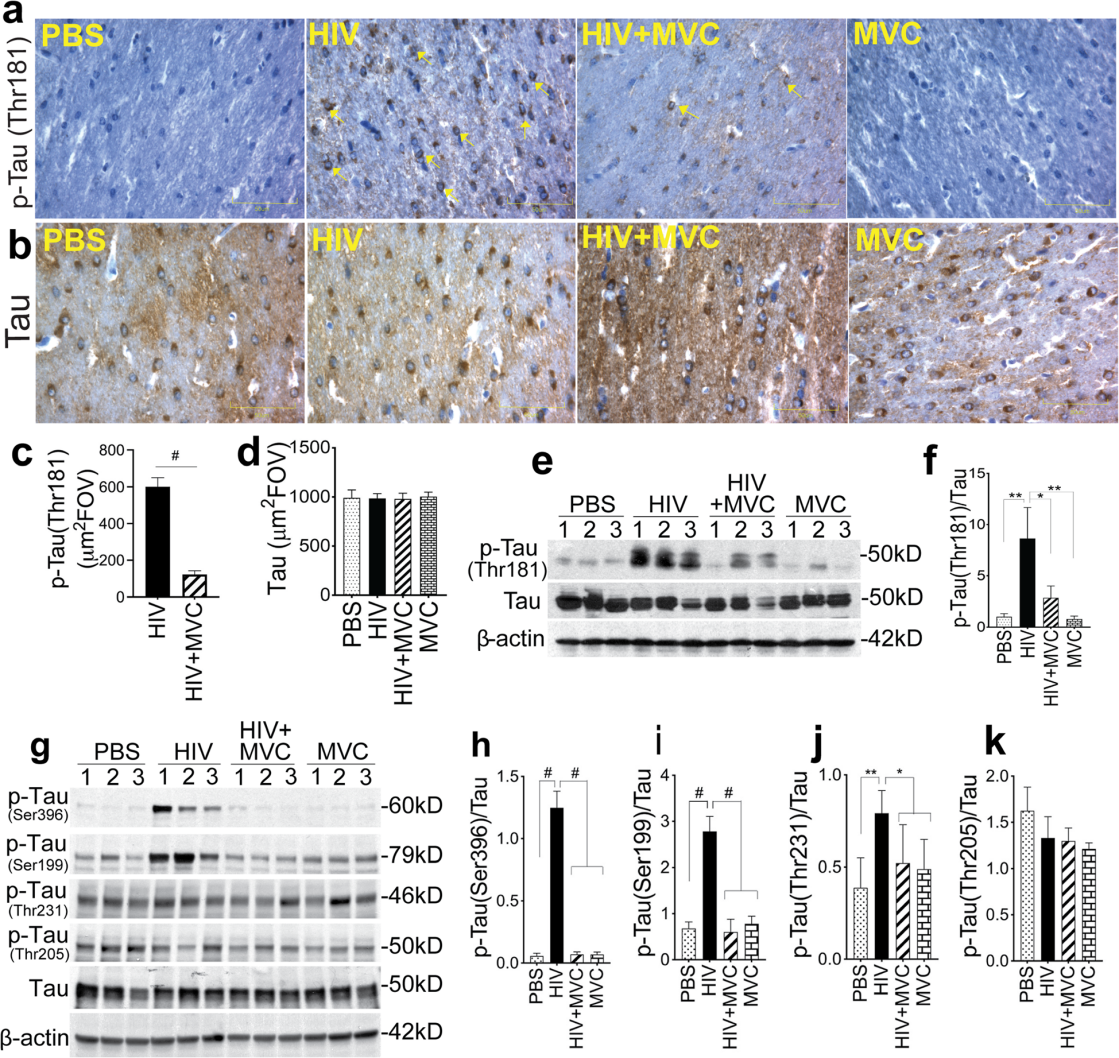
MVC abrogated HIV-induced increased Tau phosphorylation. Levels of pTau (Thr181) (**a**, **c**, **e**, **f**) and total Tau (**b**, **d**, **e**) proteins in the hippocampus fimbria of each brain sample were analyzed by immunohistochemistry (**a**, **b**) followed by densitometry quantification (**c**, **d**), as well as by Western blot (**e**) followed by densitometry quantification normalized to sample’s total Tau levels (**f**). Levels of pTau (Ser396) (**g**, **h**), pTau (Ser199) (**g**, **i**), pTau (Thr231) (**g**, **j**), and pTau (Thr205) (**g**, **k**) were also quantified by Western blot (**g**) followed by densitometry quantification normalized to sample’s total Tau levels (**h-k**). For panels **a** and **b**, images were at 40X. The four animal groups analyzed included PBS, HIV, HIV + MVC, and MVC; 9 to 11 animals in each group. ^#^P < 0.0001, **P = 0.002, *[(**f**) *P* = 0.011, (**j**) *P* = 0.048]. Error bars represent SD

Immunohistochemistry (Fig. 8a-d) and Western blot (Fig. 8e-k) analyses of brain tissues showed similar levels of total Tau protein in all animal groups. Analyses of pTau normalized to total Tau showed that HIV-1 infection increased Tau phosphorylation at Thr181 by 8.5 to 11-fold (Fig. 8e and f; *P* = 0.002), at Ser396 by 18 to 22-fold (Fig. 8g and h, P < 0.0001), at Ser199 by 3.5 to 4-fold (Fig. 8g and i, P < 0.0001), and at Thr231 by 1.6 to 2-fold (Fig. 8g and j, P = 0.002). HIV infection had no significant effect on Tau phosphorylation at Thr205 (Fig. 8g and k).

### MVC reduced HIV-induced CNS tau phosphorylation

Immunohistochemistry analysis showed that compared to infected (HIV group) animals, MVC treatment (HIV + MVC group) reduced pTau (Thr181) levels by 4.6-fold (Fig. 8a, c; P < 0.0001, df = 14, F = 5.94). Western blot analysis confirmed these findings and showed that compared to animals in the HIV group, MVC treatment of infected animals reduced pTau (Thr181), pTau (Ser396), pTau (Ser199), and pTau (Thr231) levels respectively by 3-fold (Fig. 8e and f, *P* = 0.011); 17.8-fold (Fig. 8g and h, P < 0.0001); 4.6-fold (Fig. 8g and i, P < 0.0001); and 1.5-fold (Fig. 8g and k, *P* = 0.048). MVC treatment had no significant effect on pTau (Thr205) levels (Fig. 8g and k). Two-tailed t-tests were used for Fig. 8c.

### MVC reduced Aβ retention and increased Aβ release in human MDM

Aβ produced in the CNS are cleared through phagocytosis by cells of the monocyte lineage such as macrophages and microglia [46–49]. To determine whether HIV infection and/or CCR5 inhibitors may affect this process, we quantified the uptake, retention, and release of Aβ-42 in HIV-infected and non-infected MDM, in the presence and absence of MVC. Compared to Aβ levels in non-infected MDM (526 ± 10 pg/ml), HIV-1 infection increased macrophage Aβ retention by 1.5-fold (796.5 ± 15 pg/ml) (*P* < 0.0001, Suppl Fig. 2a). MVC reduced Aβ retention by 3.4 to 4.74-fold in both infected and non-infected MDM. Aβ levels in non-infected and HIV-infected MDM exposed to MVC were 134 to 153 pg/ml and 168 to 185 pg/ml, respectively (*P* < 0.0001, Suppl Fig. 2a).

Increased Aβ retention in infected (796.5 ± 15 pg/ml) and non-infected (526 ± 10 pg/ml) MDM (Suppl Fig. 2a) correlated with minimal Aβ release in the culture media of infected (83 ± 19.6 pg/ml) and non-infected (116.6 ± 12 pg/ml) MDM (Suppl Fig. 2b). MVC increased Aβ release from non-infected and HIV-infected MDM by 5-fold and 4-fold, respectively (*P* < 0.0001, Suppl Fig. 2b). Levels of Aβ released in culture supernatants of MVC-treated non-infected and HIV-infected MDM were 575 to 608.6 pg/ml and 334 to 365 pg/ml, respectively (P < 0.0001, Suppl Fig. 2b).

### MVC increased LRP1 and decreased RAGE expression in HBMEC

Two major endothelial receptors regulate Aβ transport across the BBB: RAGE, an influx receptor that binds and transport circulating plasma Aβ into the CNS [27–30]; and LRP1, an efflux-clearance receptor that binds and transport brain-derived Aβ into the blood [31–33]. We confirmed the expression of RAGE and LRP1 in primary HBMEC (Suppl Fig. 3). Compared to controls [HBMEC treated with DMSO (vehicle)], exposure of HBMEC to Aβ-42 did not alter LRP1 or RAGE expression (Suppl Fig. 3a-c), but MVC treatment decreased RAGE expression by 2.1-fold (*P* = 0.0006, Suppl Fig. 3a and b) and increased LRP1 expression in HBMEC by 2.7-fold (*P* = 0.0002, Suppl Fig. 3a and c). In HBMEC exposed to Aβ-42, MVC also increased LRP1 (*P* = 0.03, Suppl Fig. 3a and c) and decreased RAGE expression (Suppl Fig. 3a and b) (P = 0.04).

### In the presence of MVC, LRP1 antagonist (but not RAGE antagonist) reduced endothelial Aβ uptake and retention

Quantification of Aβ-42 levels in trypsinized HBMEC (upper chamber of the transwell system) showed that in the absence of MVC, both LRP1 and RAGE inhibitors reduced endothelial Aβ uptake and retention by 2 to 2.3-fold (Suppl Fig. 3d). Compared to Aβ levels in control HBMEC (185.5 ± 20 pg/ml), Aβ levels in HBMEC treated with LRP1 and RAGE inhibitors were 79.6 ± 12 pg/ml and 84.2 ± 0.8 pg/ml, respectively (P = 0.0002, Suppl Fig. 3d). MVC alone had no significant effect on endothelial Aβ uptake [Aβ levels in MVC-treated HBMEC were 214.3 to 238 ± 28 pg/ml compared to 185.5 ± 20 pg/ml in non-MVC treated cells (Suppl Fig. 3d)]. In MVC-treated cells, RAGE inhibitors did not alter Aβ uptake and retention [Aβ levels in HBMEC treated with both MVC and RAGE inhibitors were 202 to 271.6 ± 25 pg/ml], but LRP1 inhibitors reduced Aβ uptake and retention 2 to 2.6-fold [Aβ levels in HBMEC treated with both MVC and LRP1 inhibitors were 82 to 139 ± 2.5 pg/ml, P < 0.0001, Suppl Fig. 3d].

### MVC increased transendothelial Aβ transport and LRP1 antagonist (but not RAGE antagonist) blocked MVC-induced transendothelial Aβ transport

Quantification of Aβ-42 levels in the lower chamber culture media (Aβ transported from the upper to the lower chamber of the transwell) showed that in the absence of MVC, both LRP1 and RAGE inhibitors reduced endothelial Aβ transport by 1.3 to 1.7-fold (Suppl Fig. 3e). Compared to Aβ levels in the lower chamber media of control HBMEC (105.4 ± 6.6 pg/ml), Aβ levels in lower chamber media of cells treated with LRP1 and RAGE inhibitors were respectively 59.8 ± 5.1 pg/ml (P < 0.0001, Suppl Fig. 3e) and 79 ± 11.3 pg/ml (*P* = 0.003, Suppl Fig. 3e). MVC increased Aβ transport across in vitro BBB model by 3.2 to 3.4-fold (P < 0.0001, Suppl Fig. 3e). Compared to Aβ levels in control HBMEC lower chamber media (105.4 ± 6.6 pg/ml), Aβ-42 levels in the lower chamber media of MVC-treated HBMEC were 341 to 359 ± 4 pg/ml (P < 0.0001, Suppl Fig. 3e)]. In the presence of MVC, the RAGE inhibitor had no effect on MVC-induced Aβ transendothelial transport [Aβ levels in the lower chamber media of HBMEC treated with both MVC and RAGE inhibitor were 322 to 325.5 pg/ml, compared to 341 to 359 pg/ml in MVC-treated HBMEC]. The LRP1 inhibitor reduced MVC-induced Aβ transendothelial transport by 4 to 6.24-fold [Aβ levels in the lower chamber media of HBMEC treated with both MVC and LRP1 inhibitor were 54.6 to 63 ± 3 pg/ml, compared to 341 to 359 pg/ml in MVC-treated HBMEC, P < 0.0001, Suppl Fig. 3e].

## Discussion

There is evidence of AD-like pathologies in HIV-infected individuals, including increased production of neurotoxic Aβ, Tau hyperphosphorylation, formation of amyloid plaques and NFTs-like structures in the CNS [17–20]. We have reproduced these findings in hu-PBL-NSG mice, a well-characterized and validated animal model that mimics HIV/AIDS clinical conditions [50–56]. We demonstrate that HIV-1 infection significantly increased CNS Aβ-42 and phosphorylation of Tau at Thr181, Ser396, Ser199, and Thr231 in these animals. Brain tissues from patients with AD and other dementia also showed increased accumulation of pTau in neurons, glial cells, and NFTs, including Tau hyperphosphorylation at Thr181, Thr231, Ser396, and Ser199 [57–60]. In AD mice models, brain damage is associated with increased pTau (Ser199) in the cerebral cortex and hippocampus, and reduced autophagy [61]. Our results suggest that HIV and/or viral-induced factors are directly involved in the development of AD-like pathologies in HIV-infected individuals, which agrees with previous findings. In fact, in vitro, ex vivo, and in vivo studies showed that HIV and viral proteins induced the production and aggregation of the toxic forms of Aβ (Aβ-40 and Aβ-42), the formation of amyloid plaques, and Tau hyperphosphorylation [62–68], and that Aβ peptides can further enhance HIV replication [69]. The direct role of HIV in the development of amyloidogenesis and pTau pathology is further supported by autopsy studies showing that the presence of Aβ deposits and pTau pathology in brain tissues of HIV-infected humans are often associated with high viral loads and neurocognitive impairments, including impairments in speed of information processing, attention, and working memory [21, 22]; and impairment in prospective memory [70, 71]. Studies of HIV-1 transgenic rats also showed marked increase in pTau (Thr181, Thr231, and Ser396) in the hippocampus [66]; and HIV-1 matrix protein p17 injected into mouse hippocampus co-localizes with pTau fibrils and amyloid plaques to further increase Aβ expression and induce neurocognitive impairment [67].

The current study is, to our knowledge, the first to show that HIV-induced Aβ and pTau is associated with transcriptional upregulation of GSAP, an enzyme that modulates Aβ formation [23]. In AD amyloidogenic pathway, the APP is sequentially cleaved by β and *γ*-secretases to generate neurotoxic Aβ fragments that oligomerize, form amyloid fibrils, and aggregate into amyloid plaques [72–74]. GSAP selectively and specifically regulates γ-secretase interaction with APP to increase Aβ production [23–26], and has been associated with AD and disease progression. Compared to age-matched controls, GSAP levels are significantly higher in brain tissues of AD patients [75–77], patients with other neurodegenerative diseases such as Down syndrome [78], and in AD mice models [75, 77, 79]. Silencing GSAP expression significantly reduced cellular γ-secretase activity and Aβ production in vitro [23, 80, 81]**;** reduced CNS Aβ levels, amyloid plaque formation and pTau in AD mouse models, without altering other γ-secretase function such as Notch-dependent pathways [23, 75, 77, 79–81].

Although it is well established that increased CNS and cerebrospinal fluid (CSF) Aβ-42 is a hallmark of AD pathology, there have been contradictory findings on the role of plasma Aβ-42 in AD and associated brain pathologies. Amyloidogenesis, pTau pathology, cognitive decline and AD have been associated with low/decreasing plasma Aβ-42 [82–87] and increased plasma Aβ-42 levels [88–98], whereas other studies found no significant difference in plasma Aβ of AD patients and age-matched controls [99–102], and no association between plasma and brain or CSF Aβ levels [101, 103, 104]. In our current study, HIV infection increased plasma Aβ-42 by 3-fold, compared to control groups. There have been reports of lower serum Aβ-42 in people living with HIV (PLWH) compared to gender and age matched seronegative controls [105], but HIV-infected subjects with HAND were more likely to have higher plasma Aβ-42 compared to healthy controls and PLWH without HAND [106]. This suggests that our findings of higher plasma Aβ-42 in the HIV group may be associated with higher CNS impairment. However, plasma and brain Aβ levels don’t always correlate because blood Aβ is also derived from non-CNS cells and has increased propensity to bind to other plasma proteins such as albumin, lipoproteins, and complement factors [107–110].

HIV Tat interacts with APP in vitro and in vivo to increase Aβ-42 levels and amyloid plaques [111]. Considering the direct role of HIV and viral protein in Aβ-42 production and Tau hyperphosphorylation, it would be expected that reduced viral load with ART would be associated with reduced CNS Aβ-42 and pTau; however, autopsy studies showed increased amyloidogenesis and pTau pathology in HIV-infected individuals who had been on long-term ART [19–21]. It is not known whether this is associated with specific ART regimens. Some studies showed that saquinavir and atazanavir significantly increased APP, β-secretase-1, Aβ-40, and Aβ-42 in neuronal cultures and in simian immunodeficiency virus (SIV)-infected macaques [112], and inhibited macrophages Aβ phagocytosis [113]. Other studies showed that lopinavir, nelfinavir, ritonavir, and saquinavir decreased Aβ, β-secretase-1 and γ-secretase activities in neuronal cultures but had no effect on Aβ production in APP transgenic mice [113]. The effect of CCR5 inhibitors on HIV-associated amyloidogenesis and pTau have not been investigated.

CCR5 is a major HIV co-receptor [114, 115], is expressed in many cell types [2], and the CCR5 antagonist MVC is currently used for the treatment of subjects infected with CCR5-tropic HIV [9, 10]. In our current study, MVC decreased viral loads in the blood and brain tissues and blocked HIV-induced immunosuppression. Most importantly, MVC significantly reduced HIV-induced CNS Aβ production and Tau phosphorylation, and significantly decreased HIV-induced transcriptional upregulation of GSAP, an endoprotease that catalyzes APP cleavage and Aβ formation [23]. Previous studies also showed that CCR5 was involved in neuroinflammation and cellular chemotaxis in both HAND [116–118] and AD [119–121]. CCR5 is involved in glial injury, immune dysregulation, and microglia activation in Parkinson’s disease [122] and AD [119]. MVC monotherapy reduced CNS viral loads and inflammation in SIV-infected macaques, decreased the activation of CNS leukocytes, and reduced axonal APP levels [123].

The protective effects of MVC on brain cells could be associated with its ability to cross the BBB and enter the CNS. CSF studies suggest that MVC has a high CNS penetration effectiveness score [124], with MVC levels in human CSF above the protein-adjusted inhibitory concentration (IC90) of 0.57 ng/ml [125–127]. These data would suggest that MVC readily enters the CNS. Our current study in hu-PBL-NSG mice using human equivalent doses and similar treatment schedules as in human studies showed high levels of MVC in brain tissues at 3 weeks of treatment (81–685 ng/g), and a positive correlation between plasma and brain MVC levels. The fact that there was no difference in the weight of MVC-treated and untreated animals shows that MVC did not cause overt toxicities in these animals.

Remarkably, although animals in the HIV + MVC group showed significantly reduced CNS Aβ-42 levels, they had higher plasma Aβ-42 levels (1.67-fold higher than in the HIV group and 5.7-fold higher than in the MVC group). These results suggest that in the context of HIV infection, MVC increase the transport of Aβ-42 from the brain into the peripheral blood. This hypothesis is further supported by our data showing that MVC decrease RAGE while significantly increasing LRP1 expression in BBB cells. RAGE, an influx transporter largely expressed at the BBB, binds soluble Aβ and mediate its transendothelial transport from the blood into the CNS [27–30]. Compared to age-matched controls, brain tissues from AD patients and AD animal models showed significantly higher expression of RAGE in the brain endothelium, neurons, and microglia [30, 128–130], with the highest RAGE levels correlating with higher burden of amyloid plaques and NFTs [128], impairment in learning and memory [29, 131]. LRP1, an efflux receptor expressed in BBB cells, binds, and mediates Aβ transendothelial transport from the brain into the peripheral blood [31–33]. Thus, LRP1 function as a CNS Aβ clearance receptor and our data showing that MVC decrease RAGE and increase LRP1 in HBMEC, increase transendothelial Aβ transport, and that LRP1 antagonist (but not RAGE antagonist) blocked MVC-induced transendothelial Aβ transport, suggest that MVC treatment can induce/increase CNS Aβ clearance via LRP1 pathways. Our supplemental data also showed that HIV infection increased Aβ uptake and retention and reduce Aβ release in human MDM, whereas MVC reduced Aβ retention while increasing Aβ release from MDM. This MVC-mediated Aβ efflux from both leukocytes and the CNS may have contributed to the increased plasma Aβ levels in infected MVC-treated animals.

Our data showing protective effects of MVC against HIV-induced CNS Aβ production and Tau phosphorylation suggest that an ART regimen containing CCR5 antagonists such as MVC could reduce the likelihood of HIV-induced amyloidogenesis and pTau pathology in infected individuals. This would likely be associated with improved cognition, as there is evidence that MVC-containing ART reduced leukocytes activation, reduced TNF-α, and improved neurocognitive function in some PLWH [132–135]. However, other studies of MVC effects on the CNS have reported conflicting results, with one clinical trial showing no effect of MVC-based ART on neuropsychological performance [136]; whereas another clinical trial showed that MVC-based ART marginally improved neurocognitive function but significantly improved performance in executive function [137]. The wide variations in antiretroviral drugs used in treatment regimens in these different studies likely played a role in the discrepancies observed.

Our current data also showed that HIV-induced Aβ and pTau was associated with increased neuronal injury, as evidenced by decreased expression of markers of axonal filaments (NFL), neuronal microtubules (MAP 2), and markers of neuronal development and differentiation (NeuN). This confirms previous evidence from in vitro, in vivo, and ex vivo studies showing that neuronal injury and neurodegeneration are neuropathological features of HAND [15, 138, 139] and AD [140–142]. Significantly, we demonstrated that MVC preserved neuronal structure and integrity and reduced HIV-induced downregulation of NFL, MAP 2, and NeuN. MVC also decreased HIV Tat- and V3-induced neurotoxicity [118, 143], attenuated Tat-induced neuroinflammation [118], and increased N-acetyl aspartate/creatine ratios, a marker of neuronal integrity, in PLWH [144]. MVC treatment also improved neural repair following stroke and traumatic brain injury [145]. These studies show a broad therapeutic potential of CCR5 antagonists in preventing neuronal injury and abrogating neuropathology in several CNS diseases, including HIV/AIDS.

The BBB is a complex and dynamic structure that acts as a biological interface between the blood and the brain and plays a critical role in maintaining CNS homeostasis [146, 147]. Our current data showed that in addition to increasing Aβ production and pTau, HIV infection of animals resulted in increased BBB alterations, as evidenced by decreased expression of the brain endothelial TJ proteins claudin-5, ZO-1, and ZO-2, all markers of BBB integrity. Claudin-5 is a transmembrane protein [148] whereas ZO-1 and ZO-2 are intracellular adaptor proteins [149]. Although TJ proteins such as occludin [150–152], ZO-1 [151–155], and ZO-2 [152, 154] have been detected in other cells of the neurovascular unit such as pericytes [152], astrocytes [151, 153, 154], neurons [150] and oligodendrocytes [155], these TJ proteins are primarily expressed in brain endothelial cells, the major BBB component, where they provide intercellular seals and increase paracellular tightness [156–158]. The structure and functional integrity of TJ proteins impact cellular adhesion and regulate actin cytoskeletal rearrangement and transmigration of blood leukocytes into the CNS [146, 147]. In our studies, these TJ proteins were mostly expressed on cerebral blood vessels. Our current findings are in agreement with previous studies, including human post-mortem studies, showing that both HIV and viral proteins directly induce BBB injury [11, 12, 14, 159]; and that both HAND [12, 14, 160] and AD [159, 161, 162] are associated with increased BBB injury as well as impairment in BBB tightness and function.

In addition to HIV and viral proteins directly causing BBB alterations, Aβ produced following HIV infection can further increase endothelial injury and BBB dysfunction. In fact, exposure of BBB cells to Aβ results in decreased expression of TJ proteins and increased BBB permeability [161, 163, 164]. CCR5 is also involved in this process; exposure of human brain endothelial cells to Aβ-42 or Aβ-40 induced a dose-dependent increase in CCR5 expression, chemotaxis, and monocytes transmigration through the BBB [120, 121, 165]. In this study, HIV-induced BBB alterations were associated with increased infiltration of leukocytes into the brain and significantly, we demonstrated that MVC protected the BBB and prevented HIV-induced leukocytes infiltration into the CNS. These results confirm previous in vitro findings showing that MVC and CCR5 neutralizing antibodies protect against HIV-, gp120- and Tat-induced endothelial inflammation and BBB alterations [2, 9, 37, 166].

## Conclusions

The current study is, to our knowledge, the first to demonstrate that HIV-induced Aβ and pTau is associated with transcriptional upregulation of GSAP, an endoprotease that catalyzes *γ*-secretase cleavage of APP and Aβ formation [23–26], that CCR5 is involved in HIV-induced Aβ production and Tau hyperphosphorylation in the CNS; and that the CCR5 antagonist MVC significantly reduced HIV-induced Aβ and pTau pathology, abrogates HIV-induced upregulation of GSAP, decreased RAGE and increased LRP1 expression in HBMEC, and induced/increased the transendothelial transport of Aβ via LRP1 pathways. We further demonstrate that MVC reduced HIV-induced Aβ uptake and retention while increasing Aβ release from MDM, increased plasma Aβ, reduced HIV-induced neuronal damage and BBB alterations in vivo. These results are significant and suggest that therapeutically targeting CCR5 can reduce or abrogate HIV-induced AD-like CNS pathologies. These findings have translational significance, as ART regimens containing MVC could reduce the risk of Aβ production and Tau hyperphosphorylation in the brain, increase CNS Aβ efflux, reduce brain amyloid burden, reduce HIV-induced neuronal damage and BBB alterations, and reduce the risk of AD-like CNS pathologies in infected individuals.

## Supplementary Information

**Additional file 1: Supplemental Fig. 1.** Lower magnification (4x) images show the mice brain regions analyzed. **a**: somatosensory cortex (CTX); **b**: hippocampus fimbria (Fba); MG: meningeal areas

**Additional file 2: Supplemental Fig. 2.** MVC abrogated HIV-induced increased Aβ retention and increase Aβ release in human MDM. MDM infection and Aβ treatment were performed as detailed in the Methods section. Levels of Aβ in trypsinized MDM lysates (**a**) and Aβ in MDM culture supernatant (**b**) were quantified by ELISA. Each treatment condition was performed in duplicate. ^#^*P* < 0.0001, ***P = 0.0002. Error bars represent SD

**Additional file 3: Supplemental Fig. 3.** MVC reduced RAGE and increased LRP1 expression in primary HBMEC, and increased transendothelial Aβ transport. RAGE (**a**, **b**) and LRP1 (**a**, **c**) levels in primary HBMEC were analyzed by Western blot (**a**) followed by densitometry quantification normalized to each sample’s β-actin levels (**b**, **c**). Levels of Aβ in trypsinized HBMEC lysates (upper chamber of the transwell) (**d**) and in the lower chamber culture media (**e**) were quantified by ELISA. Each treatment condition was performed in duplicate. ^#^*P* < 0.0001, ***[(**b**) *P* = 0.0006, (**c**) *P* = 0.0004, (**d**) *P* = 0.0003]; **[(**b**) *P* = 0.0015, (**e**) *P* = 0.003)]; *[(**b**) *P* = 0.04, (**c**) *P* = 0.03]. For panel **d**, *P = 0.048 compared to Aβ-exposed HBMEC not treated with MVC. “Vehicle” represents DMSO only treatment; inh: inhibitor. Error bars represent SD

**Additional file 4.** Original blots.

**Additional file 5.** Animals’ weight.

## Data Availability

All data generated or analyzed during this study are included in this publication and/or are available from the corresponding author on reasonable request.

## Abbreviations

HIV-1: Human immunodeficiency virus type-1
PLWH: people living with HIV
CCR5: C-C chemokine receptor type-5
CXCR4: C-X-C chemokine receptor type-4
MVC: Maraviroc
AD: Alzheimer’s Disease
HAND: HIV-associated neurocognitive disorders
CNS: central nervous system
NSG: NOD/scid–IL-2Rγ_c_^null^
PBL: peripheral blood lymphocytes
hu (or h): human
Aβ: amyloid-beta
pTau: phospho-Tau
APP: Amyloid precursor protein
GSAP: gamma-secretase activating protein
ZO-1: Zonula occludens-1
ZO-2: Zonula occludens-2
BBB: blood-brain barrier
TJ: tight junctions
ART: antiretroviral therapy
UNMC: University of Nebraska Medical Center
NIH: National Institute of Health
FACS: fluorescence activated cell sorting
PBS: phosphate-buffered saline
PBST: PBS containing 0.1% Tween-20
OCT: optimal cutting temperature
qPCR: quantitative polymerase chain reaction
DMSO: dimethyl sulfoxide
EDTA: ethylenediaminetetraacetic acid
ELISA: enzyme-linked immunosorbent assay
HLA: human leukocyte antigen
MAP 2: microtubule-associated protein-2
NeuN: neuronal nuclei
NFL: neurofilament-L
RT: room temperature
min: minutes
s: seconds
HRP: horseradish peroxidase
DAB: 3,3′-diaminobenzidine
DAPI: 4′,6-diamidino-2-phenylindole
FOV: fields-of-view
UPLC-MS/MS: Ultraperformance liquid chromatography-tandem mass spectrometry
IS: internal standard
RNA: ribonucleic acid
cDNA: complementary deoxyribonucleic acid
dNTP: deoxynucleoside triphosphates
LTR: long terminal repeats
pol: polymerase
Tat: transactivator of transcription
gag: group-specific antigen
GAPDH: glyceraldehyde 3-phosphate dehydrogenase
SD: standard deviation
g: grams
ng: nanograms
ml: milliliter
μl: microliter
μM: micromolar
nM: nanomolar
inh: inhibitor
MDM: monocyte-derived macrophage
HBMEC: Human brain microvascular endothelial cells
DMEM: Dulbecco’s Modified Eagle’s Medium
APP: amyloid precursor protein
RAGE: receptor for advanced glycation end products
LRP1: low-density lipoprotein receptor–related protein-1
