# Supplementary figures and images for: CCR5 antagonist reduces HIV-induced amyloidogenesis, tau pathology, neurodegeneration, and blood-brain barrier alterations in HIV-infected hu-PBL-NSG mice
Source: Mol Neurodegener. 2021 Nov 22;16:78. doi: 10.1186/s13024-021-00500-0 (PMC8607567; doi:10.1186/s13024-021-00500-0)

Supplemental Fig. 1

a

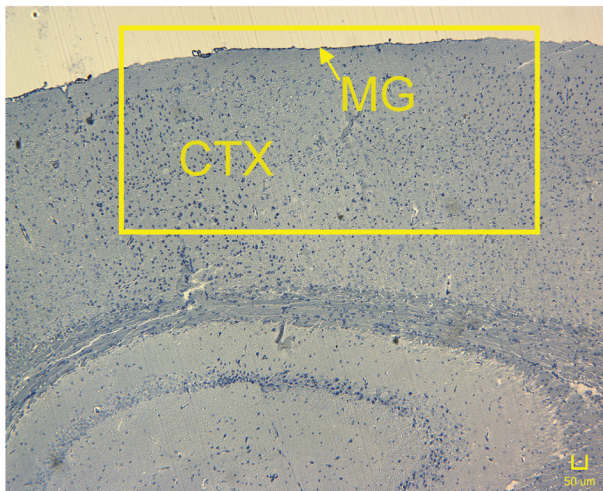

b

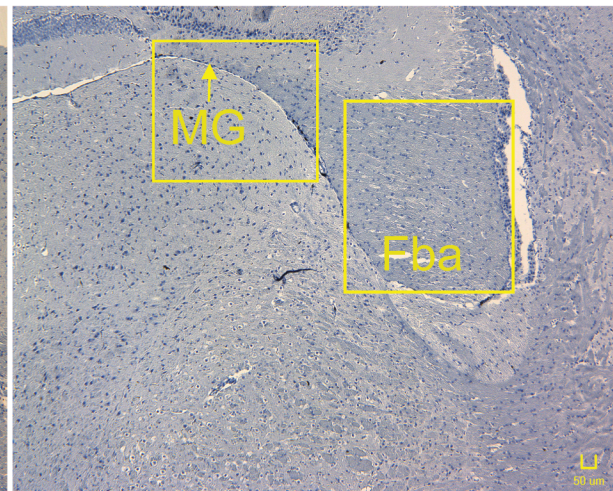

Supplement: Supplementary file 1 — Additional file 1: Supplemental Fig. 1. Lower magnification (4x) images show the mice brain regions analyzed. a: somatosensory cortex (CTX); b: hippocampus fimbria (Fba); MG: meningeal areas [file 13024_2021_500_MOESM1_ESM.pdf]

# Supplemental Fig. 2

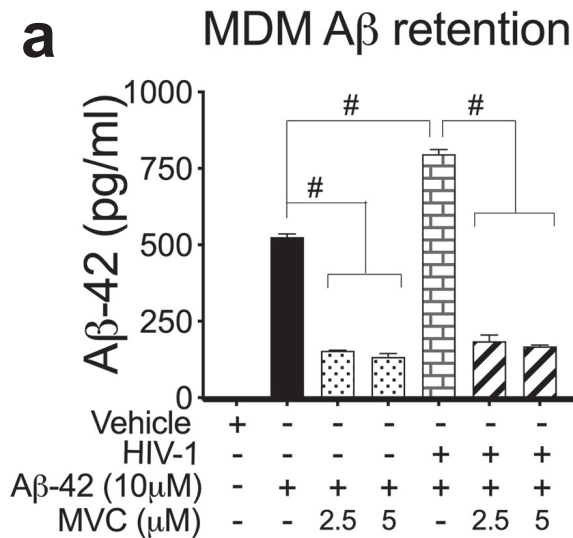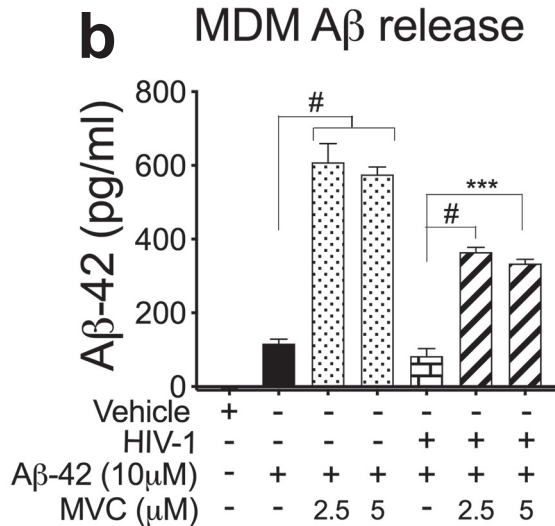

Supplement: Supplementary file 2 — Additional file 2: Supplemental Fig. 2. MVC abrogated HIV-induced increased Aβ retention and increase Aβ release in human MDM. MDM infection and Aβ treatment were performed as detailed in the Methods section. Levels of Aβ in trypsinized MDM lysates (a) and Aβ in MDM culture supernatant (b) were quantified by ELISA. Each treatment condition was performed in duplicate. #P < 0.0001, ***P = 0.0002. Error bars represent SD [file 13024_2021_500_MOESM2_ESM.pdf]

# Supplemental Fig. 3

**a**

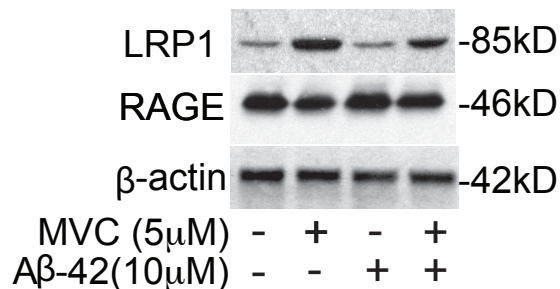

**b**

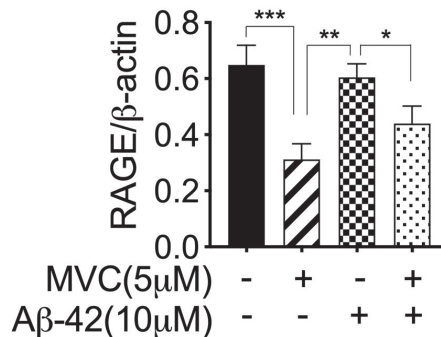

**c**

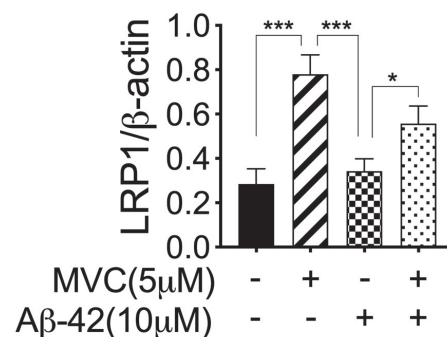

**d**

Endothelial Aβ uptake/retention

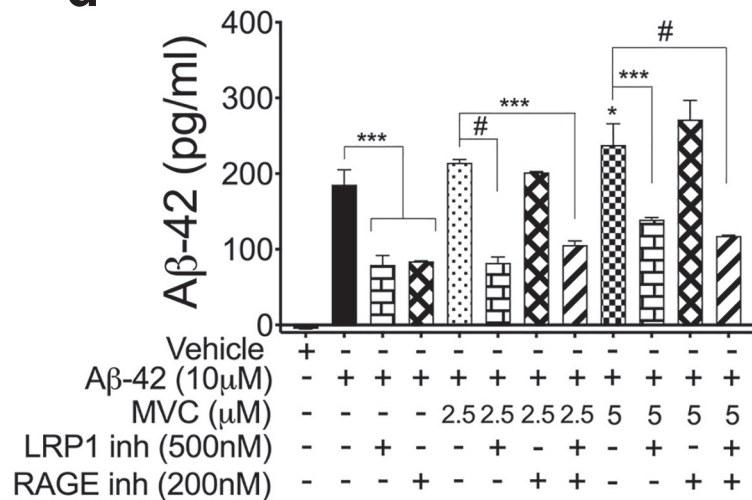

**e**

Transendothelial Aβ transport

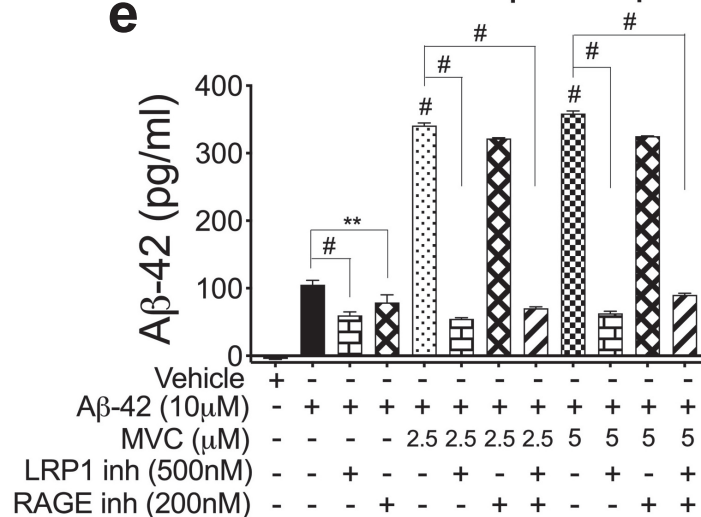

Supplement: Supplementary file 3 — Additional file 3: Supplemental Fig. 3. MVC reduced RAGE and increased LRP1 expression in primary HBMEC, and increased transendothelial Aβ transport. RAGE (a, b) and LRP1 (a, c) levels in primary HBMEC were analyzed by Western blot (a) followed by densitometry quantification normalized to each sample’s β-actin levels (b, c). Levels of Aβ in trypsinized HBMEC lysates (upper chamber of the transwell) (d) and in the lower chamber culture media (e) were quantified by ELISA. Each treatment condition was performed in duplicate. #P < 0.0001, ***[(b) P = 0.0006, (c) P = 0.0004, (d) P = 0.0003]; **[(b) P = 0.0015, (e) P = 0.003)]; *[(b) P = 0.04, (c) P = 0.03]. For panel d, *P = 0.048 compared to Aβ-exposed HBMEC not treated with MVC. “Vehicle” represents DMSO only treatment; inh: inhibitor. Error bars represent SD [file 13024_2021_500_MOESM3_ESM.pdf]
