# Supplementary material for: CCR5 antagonist reduces HIV-induced amyloidogenesis, tau pathology, neurodegeneration, and blood-brain barrier alterations in HIV-infected hu-PBL-NSG mice
Source: Mol Neurodegener. 2021 Nov 22;16:78. doi: 10.1186/s13024-021-00500-0 (PMC8607567; doi:10.1186/s13024-021-00500-0)
Supplement: Supplementary file 4 — Additional file 4. Original blots. [file 13024_2021_500_MOESM4_ESM.pdf]

Reference: Manuscript #MOND-D-21-00138R1

Figure 4c, original blots

Figure 4, panel c, Blot on
Syngene image system

Claudin-5

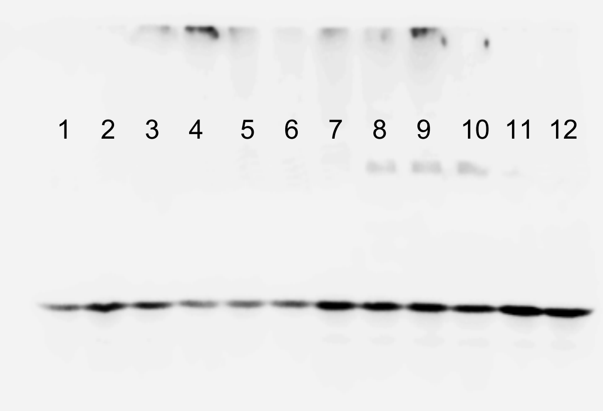

⟵ Claudin-5
(23 KD)

1,2,3: PBS
4,5,6: HIV
7,8,9: HIV+MVC
10,11,12: MVC

β-Actin

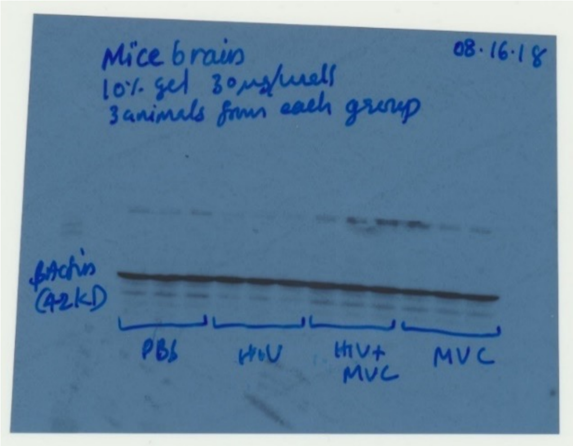

β-Actin

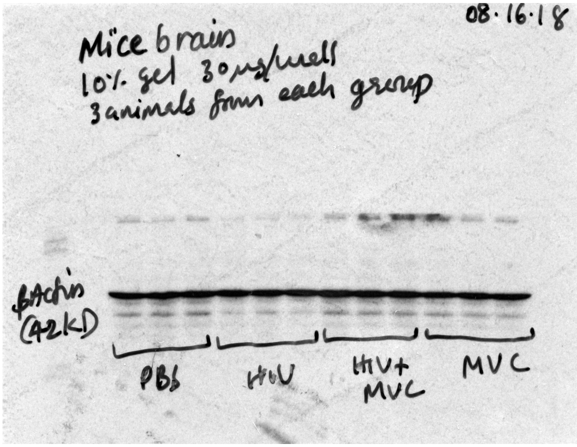

Claudin-5

β-Actin

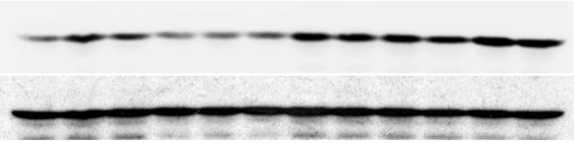

Figure 4 panel C

Figure 4g, original blots

ZO-1

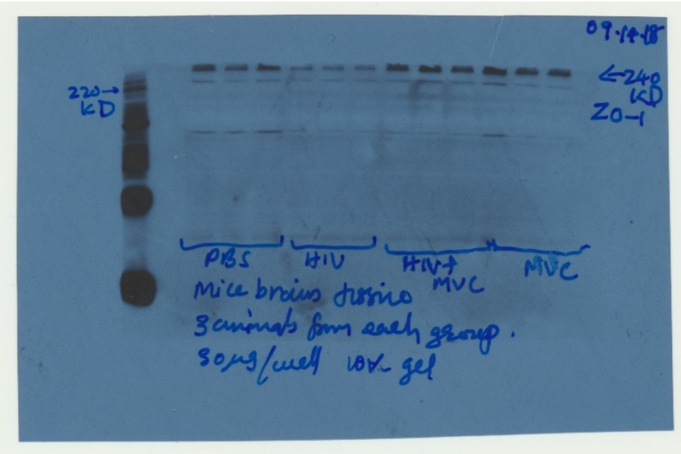

ZO-1

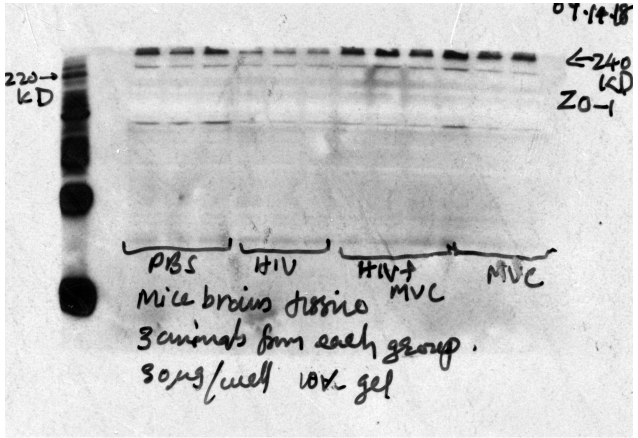

β-Actin

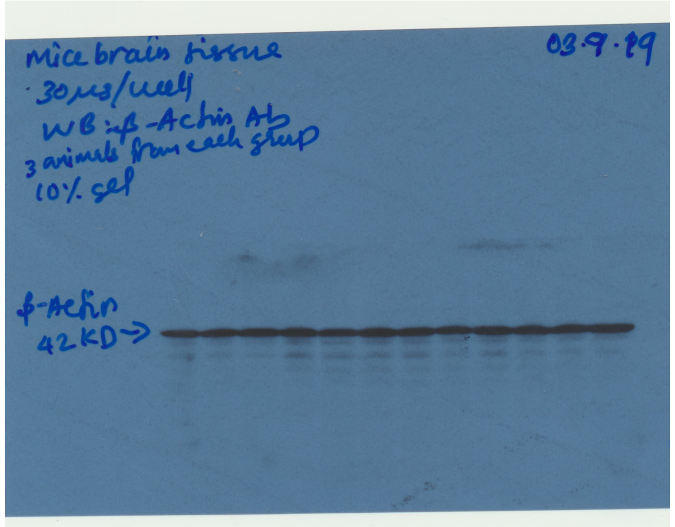

β-Actin

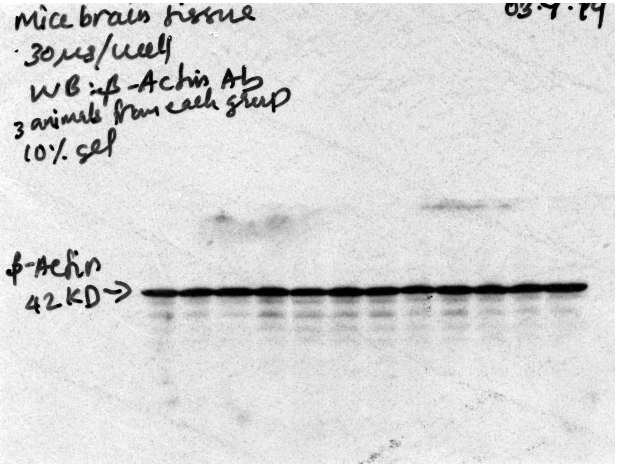

ZO-1

β-Actin

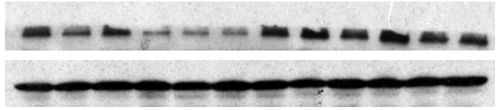

Figure 4 panel g

Figure 4k, original blots

ZO-2

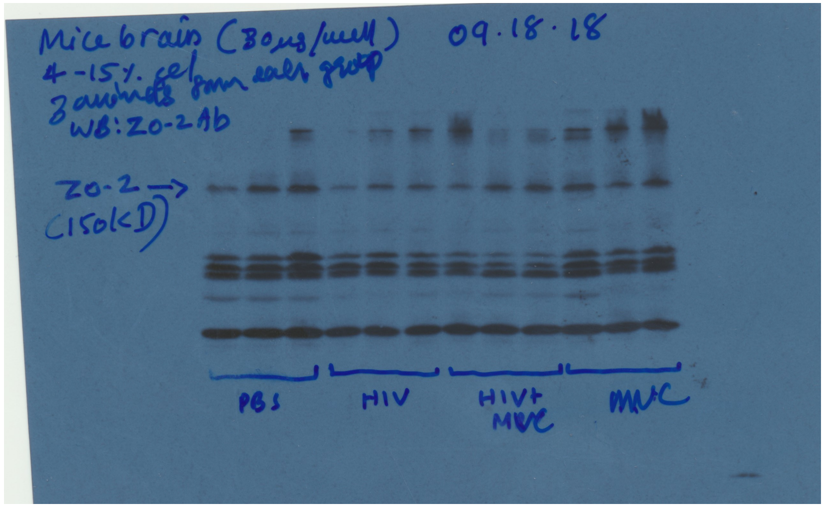

ZO-2

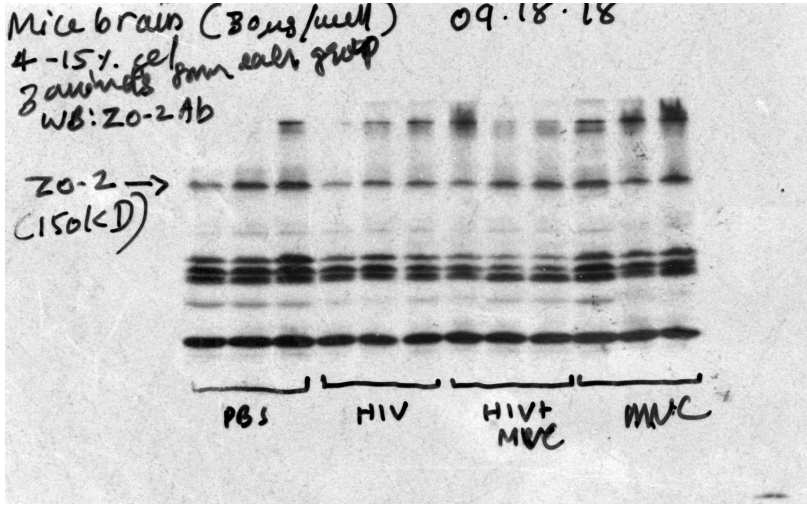

β-Actin

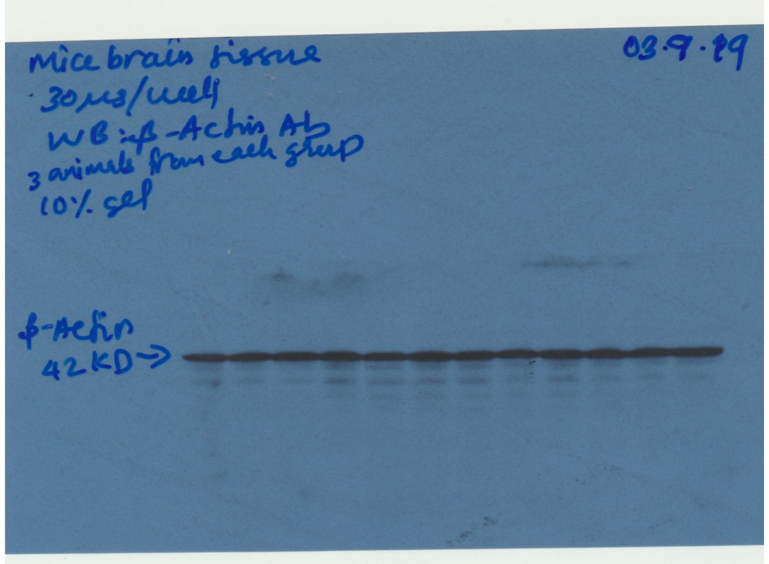

β-Actin

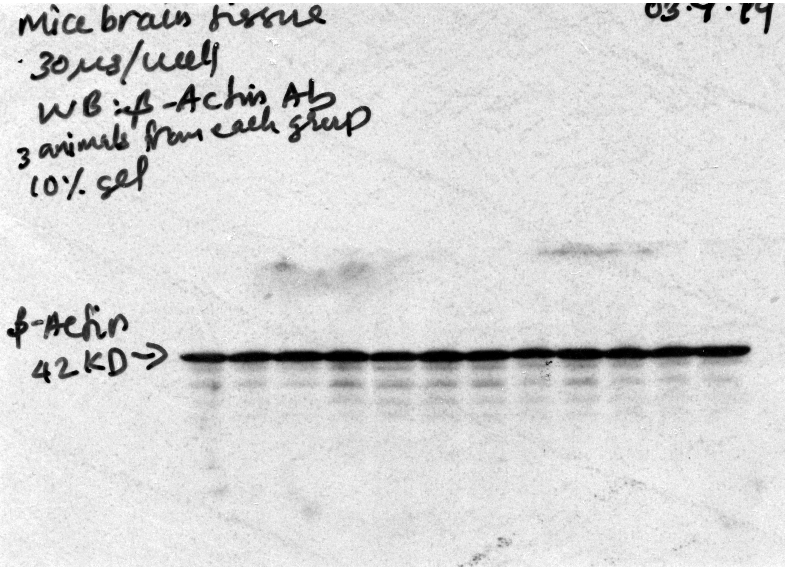

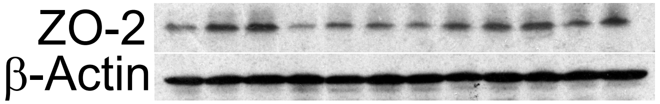

Figure 4 panel k

Figure 5c, original blots

MAP-2

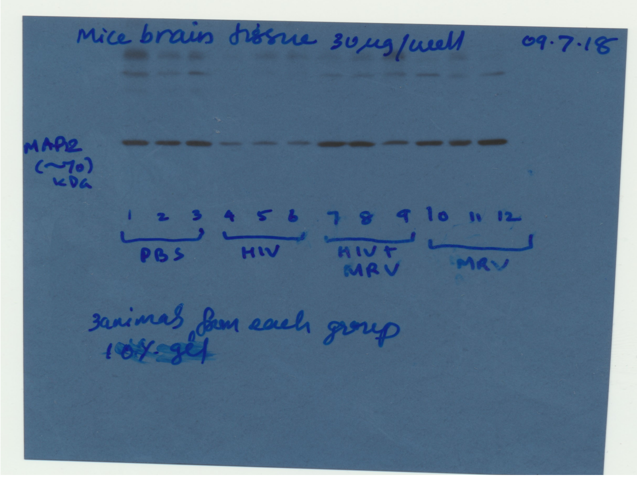

MAP-2

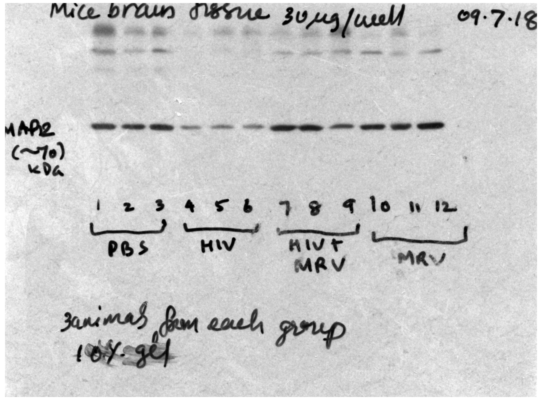

MAP-2

β-Actin

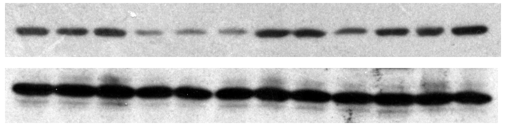

Figure 5 panel c

β-Actin

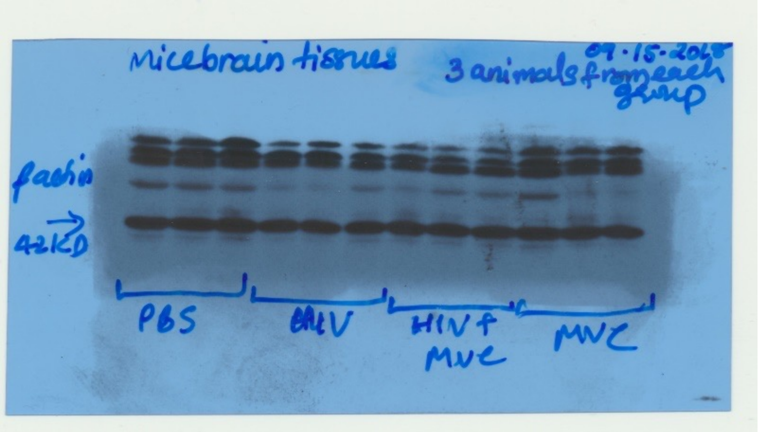

β-Actin

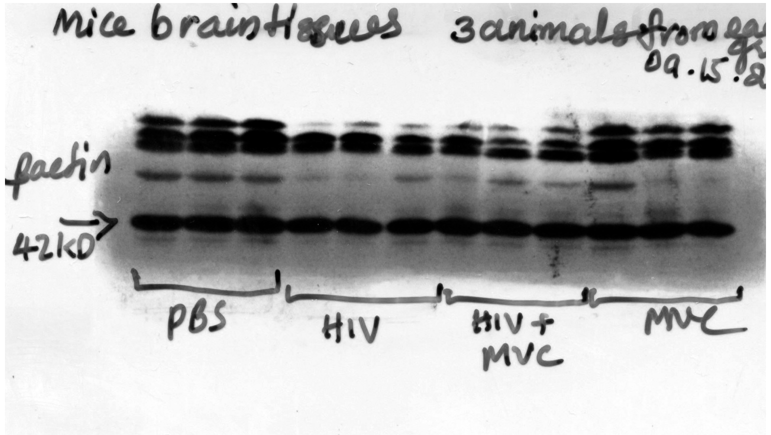

Figure 5h, original blots

NeuN

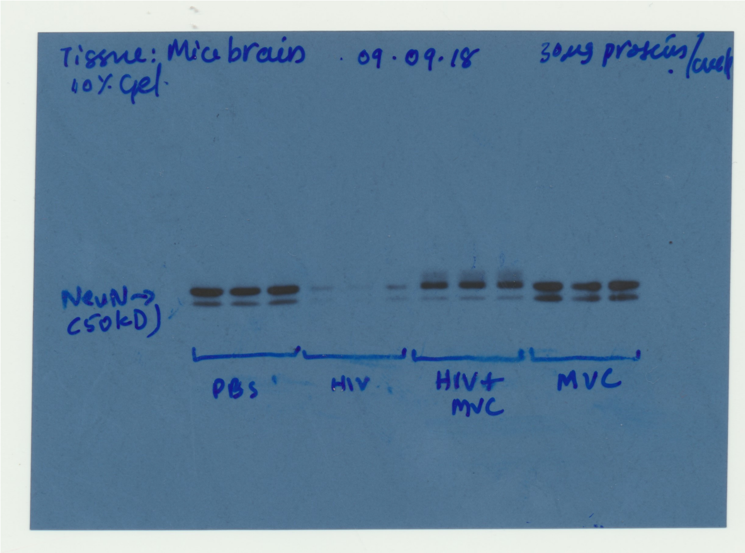

NeuN

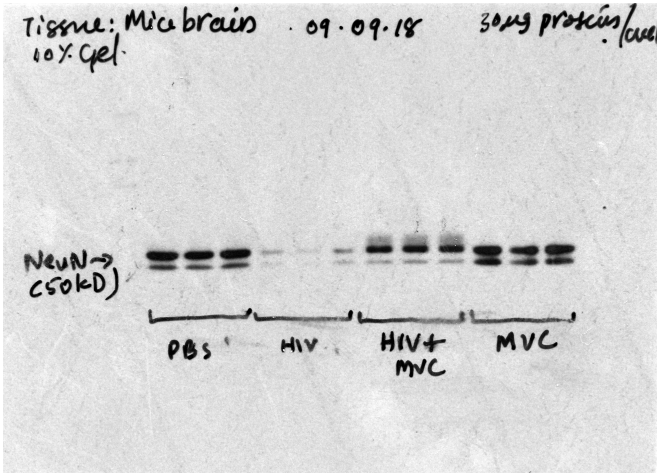

NeuN

β-Actin

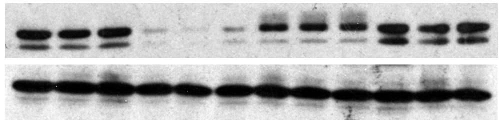

Figure 5 panel h

β-Actin

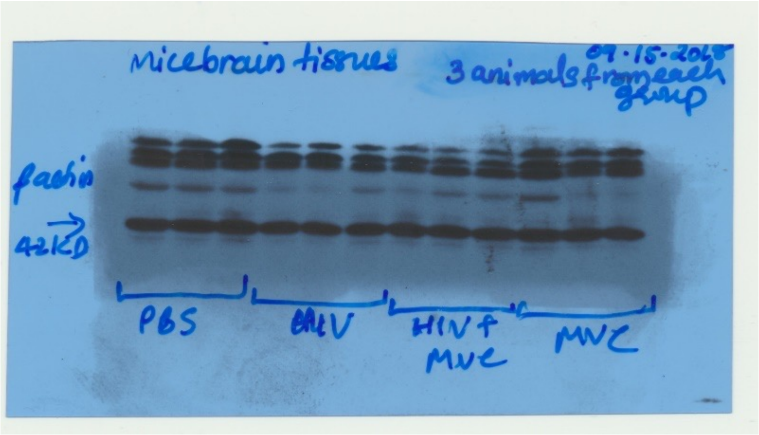

β-Actin

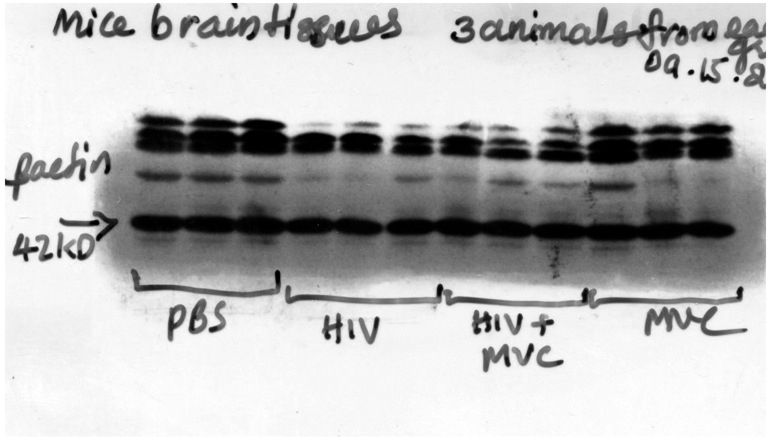

Figure 6c, original blots

NFL

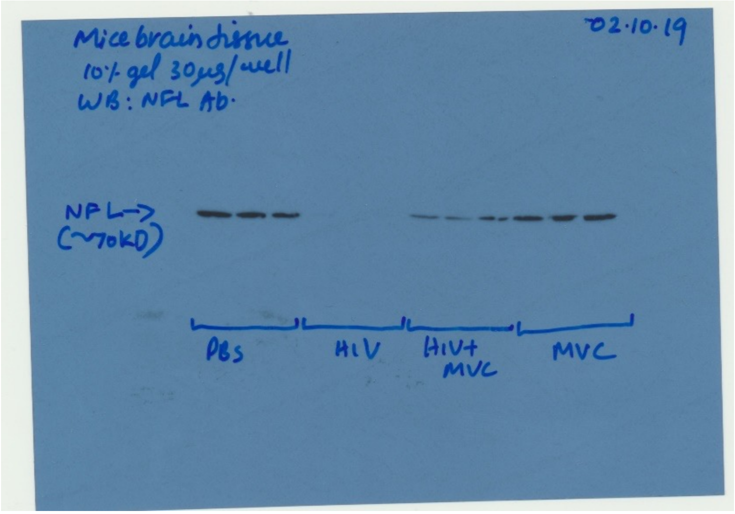

NFL

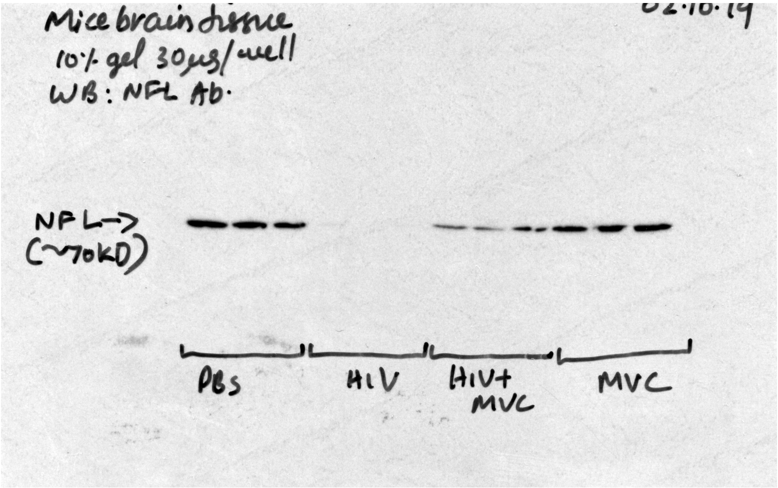

β-Actin

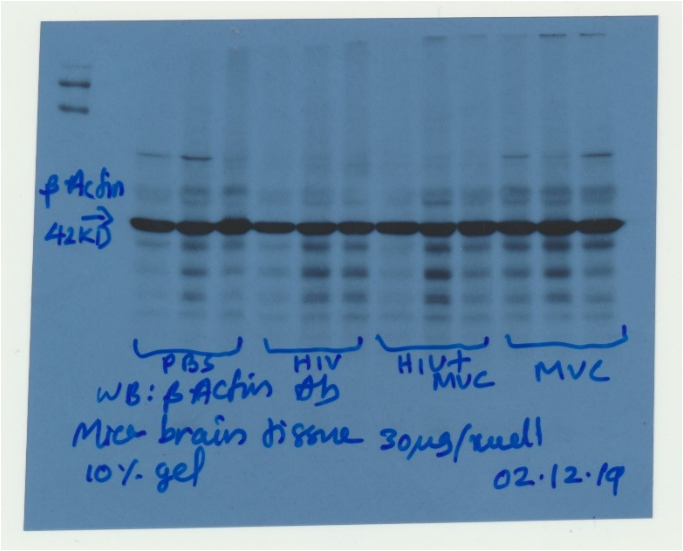

β-Actin

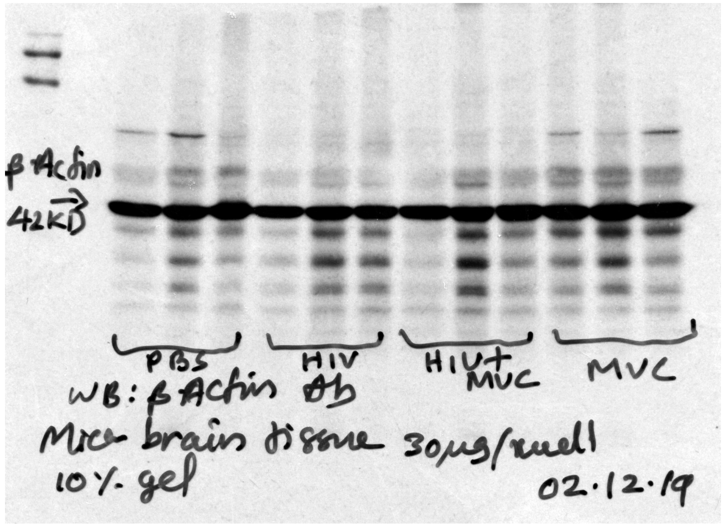

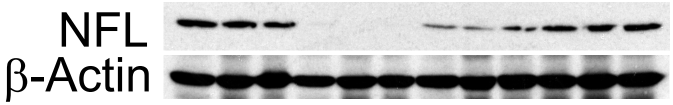

Figure 6 panel c

Figure 7d, original blots

Aβ-42

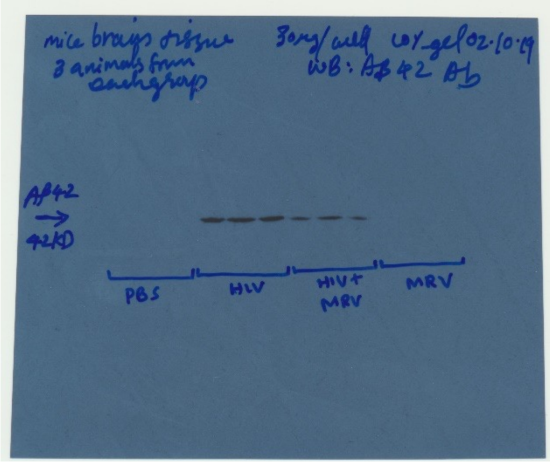

Aβ-42

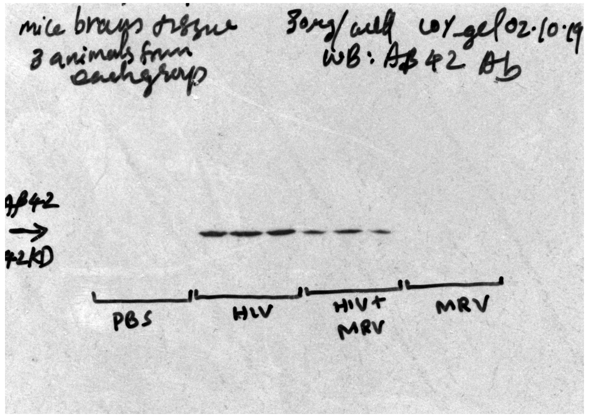

β-Actin

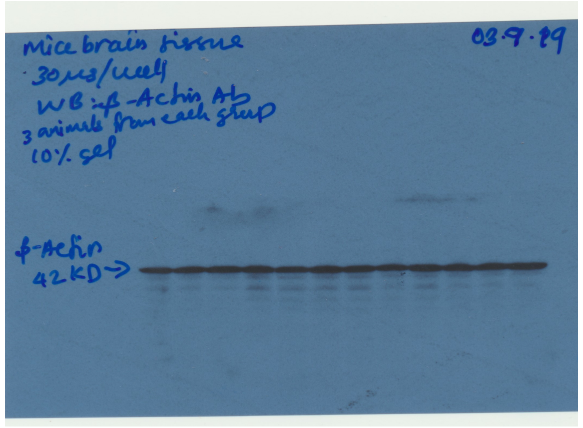

β-Actin

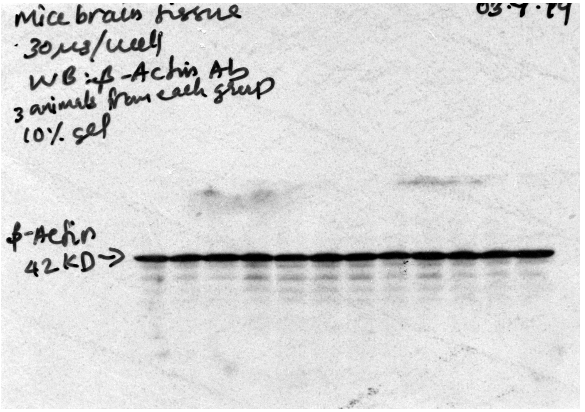

Aβ-42

β-Actin

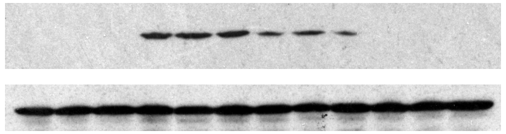

Figure 7 panel d

Figure 8e, original blots

Tau

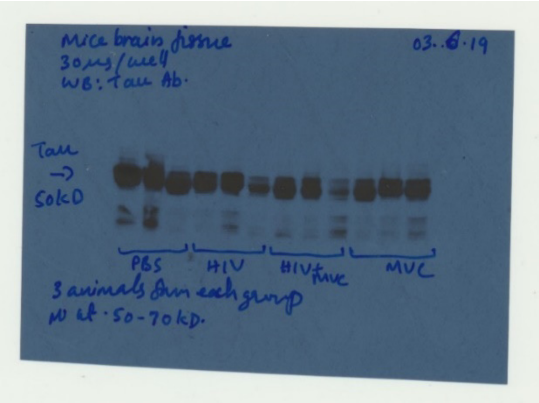

Tau

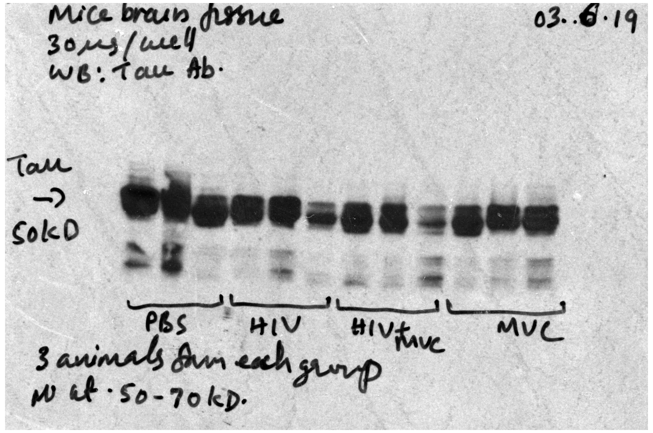

pTau (Thr181)

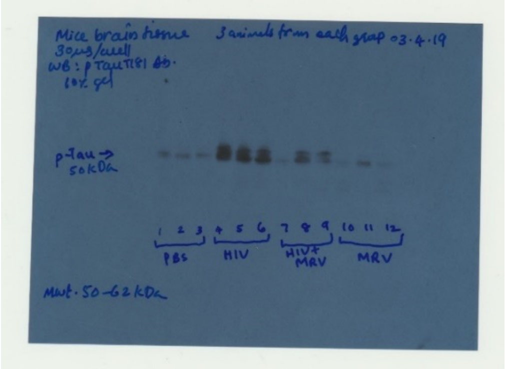

pTau (Thr181)

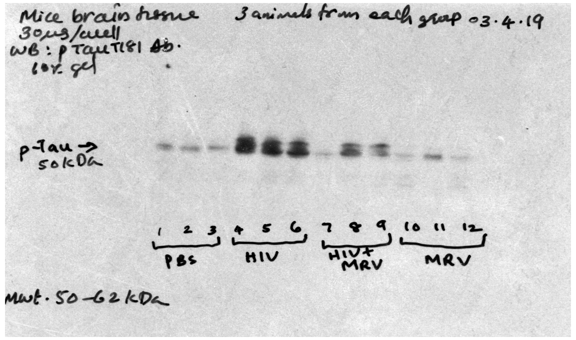

β-Actin

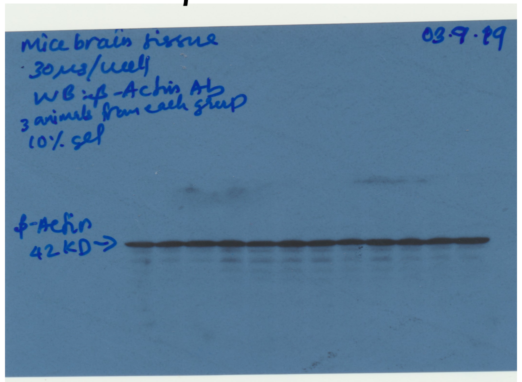

β-Actin

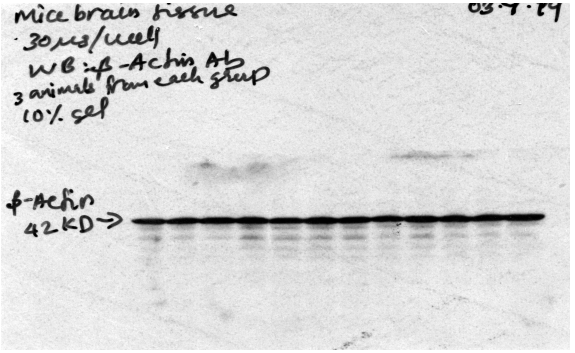

pTau (Thr181)

Tau

β-Actin

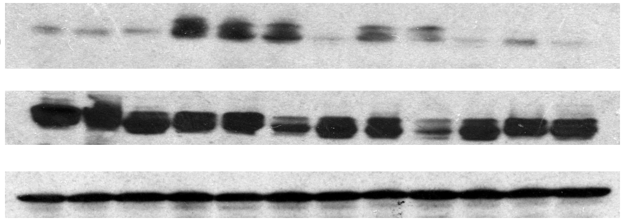

Figure 8 panel e

Figure 8g, original blots

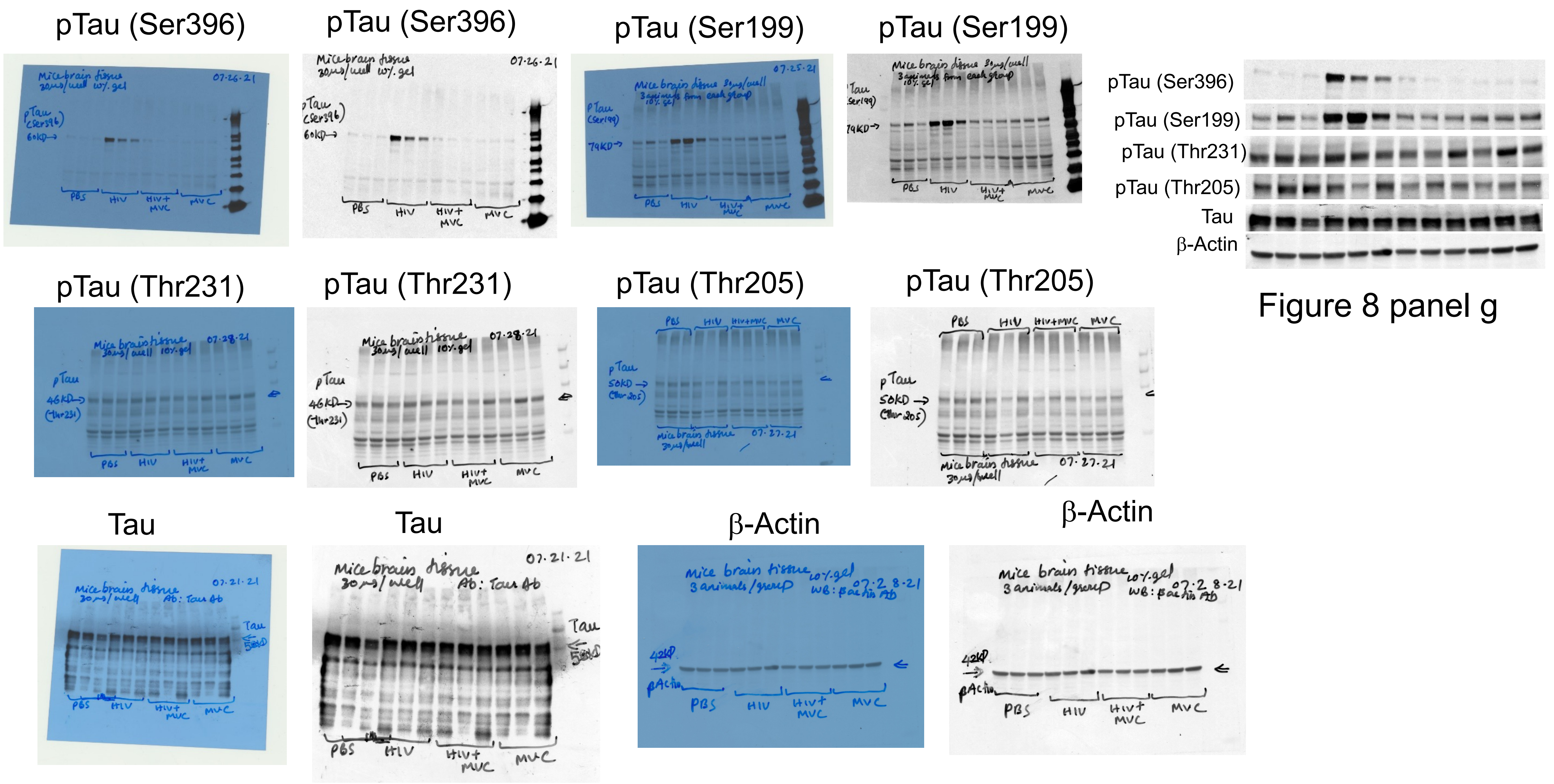

Suppl Fig.3a, original blots

LRP1

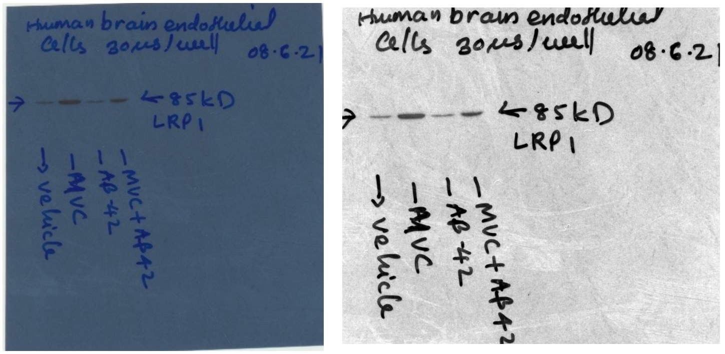

RAGE

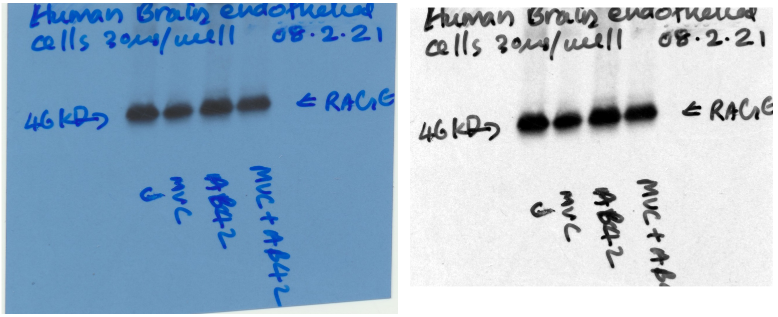

β-Actin

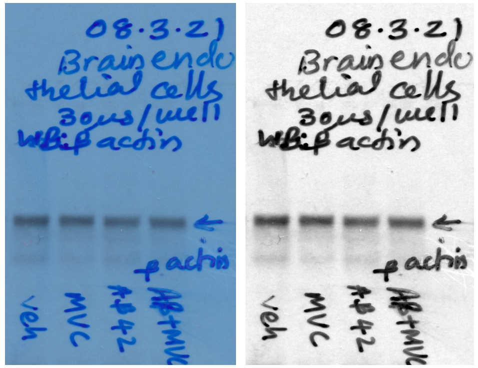

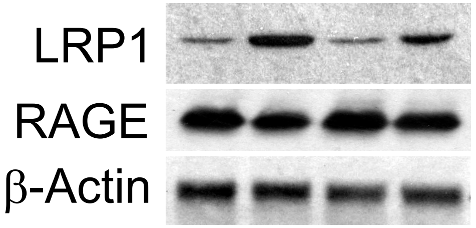

Supplemental Fig 3 panel a
