# Supplementary material for: CCR5 antagonist reduces HIV-induced amyloidogenesis, tau pathology, neurodegeneration, and blood-brain barrier alterations in HIV-infected hu-PBL-NSG mice
Source: Mol Neurodegener. 2021 Nov 22;16:78. doi: 10.1186/s13024-021-00500-0 (PMC8607567; doi:10.1186/s13024-021-00500-0)
Supplement: Supplementary file 5 — Additional file 5. Animals’ weight. [file 13024_2021_500_MOESM5_ESM.pdf]

Addional File 2

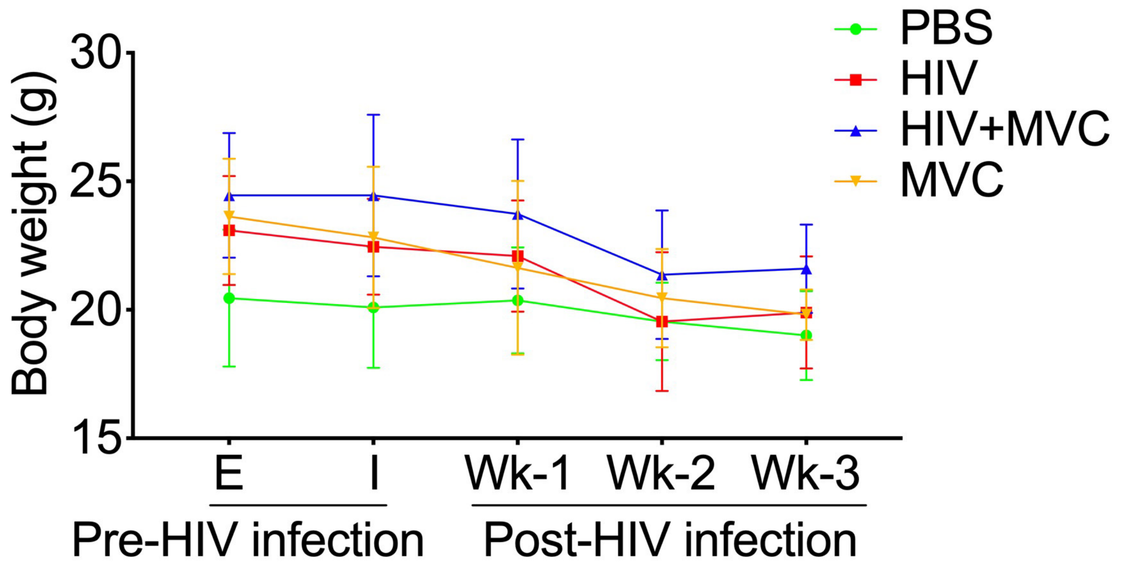

Addional File 2: Animals' weight before (pre) and after (post) infection. Data show animals' weight on the day of PBL engraftment (E), HIV-1 infection (I), and at week-1, week-2, and week-3 post-infection.
Sample size: 11 animals in each group.
